# Programmable molecular microscopy: CRISPR/Cas fluorescent probes revolutionizing spatiotemporal genomic imaging

**DOI:** 10.7150/thno.125565

**Published:** 2026-01-01

**Authors:** Xing-Yu Zhong, Yu-Xuan Yang, Yi-Fan Xiong, Gui-Chen Ye, Xi Gong, Ming-Liang Zhong, Hao-Dong He, Shao-Gang Wang, Qi-Dong Xia

**Affiliations:** Department and Institute of Urology, Tongji Hospital, Tongji Medical College, Huazhong University of Science and Technology, No. 1095 Jiefang Avenue, 430030, Wuhan, China.

**Keywords:** CRISPR/Cas, bioimaging, fluorescent probes, nucleic acid, nanotechnology

## Abstract

Bioimaging technologies visually resolve spatiotemporal dynamics of biomolecules, cells, and tissues, enabling essential insights into gene regulation, disease mechanisms, and drug metabolism. CRISPR/Cas-based fluorescent probes transform CRISPR from "genetic scissors" into "molecular microscopes," providing an indispensable tool for *in situ* decoding of molecular events in living systems. Their high nucleic acid specificity establishes CRISPR/Cas as a pivotal technology for dynamically monitoring genomic and transcriptomic events at live-cell and *in vivo* levels. This work systematically outlines design strategies and functional mechanisms of mainstream CRISPR/Cas fluorescent probes for bioimaging, encompassing five categories: fluorescent proteins, synthetic dyes, smart gated probes, nanomaterials, and multimodal integrated probes. Recent advances and persistent challenges in achieving high-sensitivity targeted imaging, effective signal amplification, and precise delivery control are comprehensively examined, including analysis of their advantages, limitations, and adaptability in complex biological environments. Building on breakthroughs in *in vivo* delivery systems, diverse carriers demonstrate significant potential for enhancing CRISPR/Cas transport efficiency, improving tissue penetration, and enabling spatiotemporal controlled release. Continued innovation drives CRISPR/Cas imaging platforms toward higher sensitivity, enhanced biocompatibility, and multifunctional integration, thereby fostering the convergence and broad application of gene editing and molecular diagnostics.

## Introduction

In medical research, bioimaging technologies are not only essential tools for elucidating molecular dynamics and spatial heterogeneity, but also serve as critical enablers for early disease diagnosis, disease progression monitoring, and therapeutic evaluation^1^, ^2^. Particularly in live-cell and *in vivo* contexts, the real-time, visual tracking of nucleic acids, proteins, and metabolic molecules has become a cornerstone of precision medicine. However, traditional imaging methods such as fluorescence *in situ* hybridization (FISH) and its derivatives typically rely on cell fixation and permeabilization processes, which not only disrupt the native structure and function of cells, but also limit their applicability in monitoring dynamic biological events^3^.

**Figure 1:**
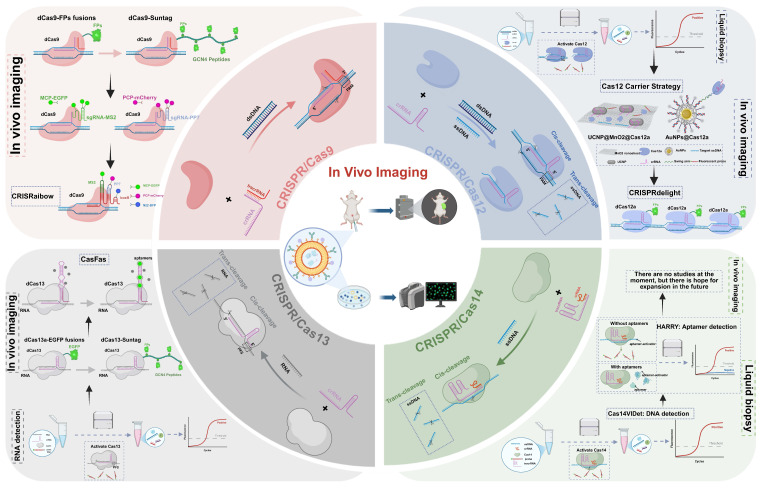
A schematic illustration of the functional transformation of the CRISPR/Cas system from basic principles to biological imaging applications. It covers four representative nucleases: Cas9, Cas12, Cas13, and Cas14. Owing to their specific recognition and cleavage capabilities, different Cas proteins have been progressively engineered into visual probes, enabling dynamic imaging of DNA, RNA, and chromatin structures. This advancement has greatly expanded the utility of CRISPR technology in gene regulation, *in vitro* diagnostics, and biological imaging.

As one of the most rapidly advancing nucleic acid tools in recent years, the Clustered Regularly Interspaced Short Palindromic Repeats (CRISPR)-Cas system has expanded beyond its original role in genome editing to encompass diverse applications in imaging, cancer therapy and diagnostics, owing to its high sequence specificity, programmability, and a wide range of effector enzymes^4^-^6^. In the field of bioimaging, CRISPR systems can be combined with fluorescent proteins (FPs), synthetic dyes, and smart-responsive modules to achieve spatiotemporal localization, dynamic tracking, and expression monitoring of both DNA and RNA (**Figure 1**)^7^, ^8^. Compared to traditional imaging techniques, CRISPR-based imaging offers advantages such as non-invasiveness and programmable targeting, enabling precise imaging in living systems and offering new insights into transcriptional regulation, cell fate determination, and disease mechanisms^9^.

More importantly, with the integration of therapeutic modules such as photosensitizers, chemotherapeutic agents, and immunoregulatory molecules, the CRISPR system has demonstrated significant potential for theranostic applications, including tumor-targeted photodynamic therapy, immune modulation, and multi-target genome editing^10^-^12^. Meanwhile, the convergence of nanomaterials and smart probes has greatly expanded its applicability within deep tissues and complex microenvironments^13^, ^14^. Against this backdrop, this review systematically summarizes the design strategies and functional mechanisms of CRISPR/Cas-based fluorescent probes, covering a broad spectrum ranging from FPs and synthetic dyes to gated probes, nanomaterials, and multimodal platforms. We highlight recent advances in imaging precision, signal amplification, and *in vivo* delivery, and further discuss the prospects of CRISPR imaging technologies in precision medicine, aiming to provide theoretical support and forward-looking insights for their interdisciplinary integration and clinical translation.

## CRISPR/Cas System: Mechanisms and Imaging Applications

The emergence of CRISPR technology has brought revolutionary changes to biological research, with its applications extending far beyond the initial scope of genome editing^15^, ^16^. By leveraging the inherent DNA and RNA targeting capabilities of various Cas proteins and engineering them into nuclease-inactivated variants (dCas)^17^-^19^, researchers have successfully developed a series of powerful molecular tools that enable the visualization of genomic loci and transcripts in living cells^20^-^22^. This chapter will systematically elaborate on the molecular mechanisms of four major Cas systems (Cas9, Cas12, Cas13, and Cas14) and their latest advances in imaging applications, with a focus on how these systems have evolved from traditional gene-editing tools into versatile, programmable molecular imaging platforms. These platforms not only support high-resolution, dynamic, and multiplexed molecular imaging but also demonstrate unique advantages and corresponding technical challenges when applied in complex intracellular environments. To provide a clear overview, we have included a comparative summary of their respective strengths and limitations for bioimaging in **Table 1**.

### CRISPR/Cas9: from a classical genome editing tool to a precision imaging platform

The CRISPR/Cas9 system was first discovered in *Streptococcus pyogenes* as an adaptive immune mechanism in prokaryotes^23^, ^24^, enabling specific defense against invading foreign DNA through recognition and cleavage^25^. Through in-depth investigation of its molecular mechanisms, the system has been successfully transformed into a highly programmable genome editing tool, rapidly becoming one of the most widely used and efficient gene editing platforms today. Its editing function relies on base-pair complementarity between the single-guide RNA (sgRNA) and the target DNA sequence, while requiring the presence of a protospacer adjacent motif (PAM; typically 5′-NGG-3′) located on the non-target (non-complementary) DNA strand, immediately adjacent to the 3′ end of the protospacer^26^, ^27^. Guided by the sgRNA, the Cas9 protein precisely recognizes the target site and induces a double-strand break in the DNA, triggering the cell's own DNA repair mechanisms to achieve precise genome modification and functional reprogramming^24^, ^28^, ^29^. Beyond its traditional gene editing function, the CRISPR/Cas9 system has been extended to non-destructive gene regulation applications^30^. By introducing point mutations in the two catalytic domains (HNH and RuvC), a catalytically inactive dCas9 protein that retains DNA recognition ability but lacks cleavage activity can be obtained^31^. Since then, dCas9 has been widely used to construct live-cell chromosome visualization systems, opening a new avenue for genome “labeling without disruption”.

The initial dCas9 imaging strategies were mostly based on direct fusion with FPs, such as green fluorescent protein (GFP) and mCherry, or utilized nanobody conjugation techniques to achieve fluorescent labeling of repetitive sequences in live cells^7^, ^32^. However, when targeting non-repetitive sequence sites, these systems often suffer from low signal-to-noise ratios due to the limited copy number of fluorescent signals, which restricts imaging sensitivity and resolution. To address this issue, researchers introduced a signal amplification strategy at the protein level. A representative system is SunTag, which consists of a series of tandem peptide repeats that can be specifically recognized by antibody-fluorescent protein fusion constructs. By fusing multiple tandem peptide repeats to the C-terminus of dCas9, the system recruits multiple copies of FPs, thereby achieving cascade amplification of the signal and effectively enhancing the imaging signal-to-noise ratio. This approach is particularly suitable for detecting low-abundance targets or weak-signal regions, achieving up to 24-fold signal amplification^33^, ^34^. However, extensive modifications at the protein level may adversely affect the targeting efficiency and structural stability of dCas9. To circumvent these limitations, signal amplification strategies have progressively transitioned to the RNA level, giving rise to a variety of engineered sgRNA tagging systems. These systems incorporate artificial hairpin structures, such as MS2 and PP7, at the 3' terminus of sgRNA, and are co-expressed with cognate RNA-binding protein-fluorescent protein fusion constructs. This design facilitates the local enrichment of fluorescent signal clusters at genomic target sites, thereby achieving robust signal amplification while effectively minimizing background interference^35^, ^36^. Moreover, multicolor and multiplexed imaging approaches are increasingly being recognized as emerging frontiers in this domain. Among these, the CRISPRainbow system represents a significant advancement in the field. By simultaneously incorporating multiple RNA tags, it enables dynamic imaging of multiple loci with diverse colors and across various spatial and temporal scales, providing a direct visualization tool for studying genome folding, chromatin interactions, and transcriptional regulation^20^. Consequently, Cas9 has evolved from a simple gene-editing tool into a highly compatible, modular, and extensible nucleic acid-targeting platform, laying a programmable foundation for the development of subsequent probe systems and switch control modules.

### CRISPR/Cas12: a signal-amplifying imaging tool based on ssDNA cleavage

The CRISPR/Cas12 system is one of the nucleases extensively studied following Cas9, with representative members including Cas12a (Cpf1) and Cas12b (C2c1), which are widely distributed across various bacterial species^37^-^39^. This system belongs to the Class 2, Type V CRISPR systems, and its recognition mechanism relies on base-pair complementarity between the crRNA and the target DNA, with a thymine-rich PAM sequence located on the non-target DNA strand, immediately adjacent to the 5′ end of the protospacer^40^. Compared to the Cas9 system, Cas12 exhibits significant differences in both structure and function. Cas12 has a simpler molecular structure and does not require trans-activating CRISPR RNA for assistance. Upon binding to the target DNA, it not only performs precise cleavage but also activates efficient, non-specific cleavage of nearby single-stranded DNA (ssDNA), producing a “collateral cleavage” effect^37^, ^41^. This cleavage property makes Cas12 a highly advantageous tool for signal amplification and visualization detection. Especially *in vitro* molecular diagnostics, Cas12 can amplify a single recognition event into multiple signal releases by activating continuous cleavage of ssDNA probes after detecting trace amounts of target DNA^42^, ^43^. Commonly used probes typically carry both a fluorophore and a quencher. Fluorescence is suppressed before cleavage and released upon activation, enabling the construction of sensitive, low-background detection systems. This strategy offers rapid response and is particularly suitable for detecting low-abundance nucleic acids or trace samples. Based on this principle, various rapid nucleic acid detection tools have been developed, such as SHERLOCK^44^ and DETECTR^37^, which are widely applied for the rapid detection of specific pathogens in clinical samples. Furthermore, integrating CRISPR amplification platforms with technologies such as microspheres and microfluidics enables highly sensitive and portable diagnostics for target analytes, demonstrating broad application potential^45^-^47^.

However, the CRISPR/Cas12 system is limited by interference from the complex intracellular environment, which compromises its enzymatic activity and renders its trans-cleavage function fragile and unstable, posing significant challenges for imaging applications under cellular conditions. To expand the utility of Cas12 for *in vivo* imaging, current research is focusing on enabling controlled activation of its trans-cleavage activity within cells. To overcome this bottleneck and achieve high-sensitivity and high-specificity intracellular imaging, two primary strategies have been adopted: on one hand, protective carriers based on nanomaterials are being developed to shield Cas12 proteins from intracellular inhibitory factors, thereby enhancing the stability and durability of their activity; on the other hand, spatiotemporal control of Cas12 activation is achieved using gated mechanisms such as light-responsive or chemically inducible switches, which help minimize nonspecific cleavage and background noise^14^, ^48^. Additionally, since Cas12 possesses intrinsic cis-cleavage activity toward DNA, its direct application in *in vivo* imaging may pose a potential risk of genomic damage. To address this concern, researchers have explored its capability for RNA recognition and detection, and have successfully developed Cas12a-based RNA imaging platforms^49^, thereby expanding the scope of Cas12 applications in live-cell imaging. On the other hand, catalytically inactive Cas12 (dCas12) has emerged as a promising alternative by retaining DNA-binding capacity without cleavage activity. Leveraging this feature, Yang *et al.* developed an innovative platform known as CRISPRdelight^21^. By employing an optimized CRISPR array to enable the design of multiple crRNAs, this system precisely targets multiple DNA regions, thereby enhancing signal enrichment while avoiding genomic damage and improving imaging safety.

In summary, although the application of the Cas12 system in complex intracellular environments still faces considerable challenges, the integration of materials engineering, optogenetic strategies, and enzymatic activity regulation holds great promise for establishing Cas12 as a powerful tool in live-cell imaging. These advances are expected to drive molecular imaging technologies toward higher sensitivity and specificity.

### CRISPR/Cas13: an RNA-targeted platform for dynamic molecular imaging

CRISPR/Cas13 is one of the few CRISPR systems that directly targets RNA. It belongs to the Class 2, Type VI CRISPR system and primarily includes four subtypes: Cas13a, Cas13b, Cas13c, and Cas13d^50^-^53^. Unlike the DNA-targeting nature of CRISPR/Cas9, CRISPR/Cas12, and CRISPR/Cas14 systems, the CRISPR/Cas13 system is guided by crRNA to recognize and cleave specific single-stranded RNA sequences bearing a protospacer flanking site (PFS), thereby enabling RNA-level regulation^52^-^54^. This unique property endows Cas13 with distinct advantages in areas such as functional analysis of non-coding RNAs and intracellular RNA imaging. In addition to its RNA-targeting capability, Cas13 also exhibits a “collateral cleavage” effect similar to that of Cas12. Upon binding to the target RNA, its enzymatic activity is activated, leading to nonspecific cleavage of surrounding single-stranded RNAs (ssRNAs)^51^ This property has been harnessed in the development of the SHERLOCK system^55^ for rapid viral nucleic acid detection. While this effect offers significant advantages for *in vitro* molecular diagnostics, it poses potential cytotoxicity and background noise issues in intracellular imaging applications. To overcome these limitations, researchers have generated catalytically inactive Cas13 variants (dCas13) by introducing mutations into the HEPN nuclease domain, thereby retaining RNA-binding capability while eliminating endonuclease activity^50^, ^56^, ^57^. dCas13 not only significantly reduces cytotoxicity but can also be functionally fused with fluorescent modules^19^, ^58^, laying a technical foundation for high spatiotemporal resolution RNA visualization and dynamic tracking.

RNA imaging systems based on dCas13 have been successfully applied in various cellular models and are gradually becoming important tools for visualizing RNA in living cells. Early studies demonstrated that dPspCas13b, a subtype of Cas13b, exhibits high efficiency and specificity in labeling endogenous RNAs^58^. Compared to other Cas13 variants, it offers superior binding stability and a smaller molecular size, making it well-suited for fusion with FPs to enable spatial localization and dynamic tracking of intracellular RNAs^19^. Compared to conventional FISH methods^3^, the dCas13-based imaging system does not require cell fixation and enables real-time tracking of RNA expression, transport, and degradation, offering a new technological avenue for studying RNA metabolism. However, early versions of the dCas13a system exhibited limitations in signal intensity and background suppression, with insufficient sensitivity for detecting low-abundance transcripts. To enhance imaging signals, various amplification strategies have recently been integrated into the dCas13 platform to improve its detection performance. Chen *et al.* incorporated the SunTag module into the dCas13a system by appending tandem repeat structures to the protein terminus, thereby enriching FPs and significantly enhancing imaging sensitivity and spatial resolution^59^. For multiplexed imaging, Cas13b has become the preferred tool due to its compact structure and high binding stability, making it well-suited for multicolor and multichannel RNA visualization. Based on dPspCas13b, Tang *et al.* optimized the sgRNA structure and introduced multiple fluorescent tags to develop the CasFAS system, which enabled efficient imaging of endogenous RNA loci^22^. This system markedly improved spatial resolution and imaging throughput, providing a more precise and versatile platform for investigating RNA localization, expression regulation, and dynamic changes at the subcellular level during development and disease progression.

Overall, the Cas13 system, particularly imaging platforms based on dCas13b, is gradually replacing traditional fixed-cell detection methods. With its advantages of reversibility, non-invasiveness, and high specificity, it is propelling live-cell RNA imaging technologies toward more advanced and multidimensional applications.

### CRISPR/Cas14: prospects of ultra-compact cas proteins for *in vivo* delivery and microenvironment imaging

Cas14, also classified as Cas12f, is an ultra-compact effector protein discovered within the CRISPR/Cas system. It was first identified by scientists in the DPANN superphylum of symbiotic archaea^60^. Compared to conventional Cas9 and Cas12 systems, Cas14 has a significantly smaller molecular weight and consists of only a single RuvC nuclease domain, resulting in an exceptionally compact structure that confers unique functional advantages. Cas14 preferentially cleaves ssDNA with minimal or no dependence on PAM sequences^60^, providing flexibility in target recognition. In contrast, its activity on double-stranded DNA (dsDNA) is low and only modestly triggered by 5' T- or C-containing PAMs, distinguishing it from Cas12 nucleases^61^, ^62^. This combination of relaxed PAM requirements and high ssDNA specificity makes Cas14 particularly suitable for precise targeting of non-coding regions, viral DNA, and low-complexity sequences. Based on this characteristic, Lai *et al.* developed the Cas14VIDet platform, which successfully enabled highly sensitive detection of antibiotic resistance genes in *Helicobacter pylori*^63^. Similar to other members of the Cas12 family, Cas14 exhibits trans-cleavage activity toward non-specific single-stranded ssDNA upon recognizing its complementary target DNA^64^. This mechanism offers a natural advantage for signal amplification and multiplex target detection^63^, ^65^. Therefore, Cas14 not only possesses precise target-cleaving capability but also shows great potential for applications in nucleic acid diagnostics, genetic testing, and biosensing.

With its ultra-small molecular size and highly programmable targeting capability, Cas14 holds great promise for future applications in CRISPR-mediated live-cell imaging. Its dual recognition ability for both ssDNA and dsDNA, along with minimal dependence on PAM sequences, enables the potential for multiplex, dynamic, and high-resolution imaging within living cells. In particular, Cas14 offers inherent technical advantages in multiplex detection, signal amplification, and low-background imaging, making it a strong candidate to advance CRISPR-based imaging technologies toward higher spatial precision and enhanced sensitivity.

## Fluorescent Probes in CRISPR/Cas Systems: From Signal Generation to *In Vivo* Imaging

With the advancement of CRISPR/Cas systems in molecular imaging, fluorescent probes for *in vivo* imaging are evolving from traditional to intelligent designs and from single-mode to multimodal platforms. Based on their properties and integration with the Cas system, current CRISPR/Cas probes can be categorized into five types: fluorescent protein probes, synthetic fluorescent dyes, smart gated probes, nanomaterial-based probes, and multimodal fusion probes. As systematically quantified in **Table 2**, each type presents a unique combination of sensitivity, signal-to-noise ratio, photostability, and cytotoxicity, thereby defining their respective niches in practical imaging applications.

### Fluorescent protein probes: a fundamental and classic imaging approach

FPs were among the first signaling molecules developed for *in vivo* imaging, and their discovery and evolution represent a milestone in molecular imaging technology. In 1962, Shimomura and colleagues first extracted and identified the GFP from the jellyfish *Aequorea victoria*^66^. Subsequently, scientists such as Chalfie and Tsien achieved heterologous expression and structural optimization of GFP in mammalian cells, laying the foundation for the widespread application of FPs^67^, ^68^. With the continuous advancement of molecular engineering and directed protein evolution, the in-depth understanding of the GFP crystal structure has paved the way for its rational design^69^, leading to the development of various spectral variants and functionally enhanced proteins, including enhanced GFP^70^, yellow fluorescent protein^71^, and cyan fluorescent protein^72^. It is important to note, however, that most red-shifted FPs (such as mCherry and mRFP1)^73^, ^74^ were not derived from the gradual engineering of GFP, but originated from the discovery of novel natural FPs in corals and other marine organisms^75^. These variants not only cover a full emission spectrum from blue to red but also exhibit significant improvements in brightness, photostability, pH tolerance, and refractive index, providing a solid technical foundation for multi-channel, long-term, and high-resolution imaging^76^-^78^.

**Figure 2:**
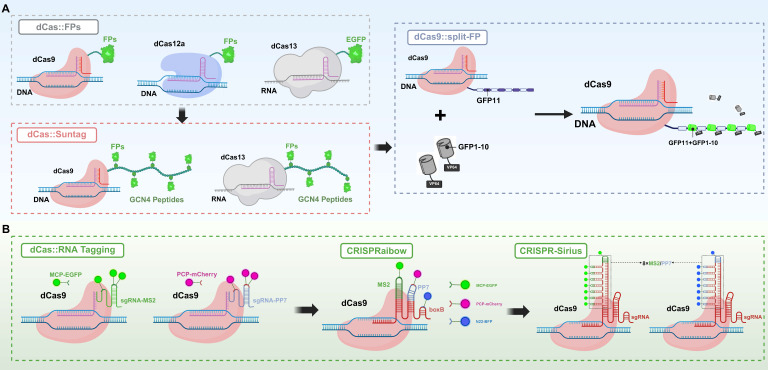
FPs-based imaging strategies enabled by CRISPR/Cas systems. This figure illustrates two major strategies for incorporating FPs into CRISPR-based bioimaging platforms, highlighting their mechanisms of action and labeling schemes. (A) Direct FP fusion with Cas proteins: Fluorescent proteins are directly fused to catalytically dCas variants to enable visualization of specific DNA or RNA targets. This includes single FP fusion and amplified systems like SunTag, where multiple GCN4 peptides recruit multiple FPs for signal enhancement. Another approach is the split-FP strategy, where dCas9 is fused with a GFP11 fragment and complemented by the GFP1-10 domain expressed separately, enabling conditional assembly of a complete fluorescent signal at the target locus. (B) FP labeling via sgRNA scaffolds: This strategy leverages engineered sgRNA that include RNA aptamer loops, which recruit FP-conjugated RNA-binding proteins. The CRISPRainbow system implements this approach to enable multicolor labeling of genomic loci by combining different aptamer-protein pairs within a single sgRNA scaffold. Building upon this, the CRISPR-Sirius system introduces multiple tandem copies of aptamers (MS2/PP7) into the sgRNA, thereby enhancing fluorescence intensity by recruiting more FP-tagged proteins at the target site. Together, these systems allow for multiplexed, dynamic, and high-signal imaging of chromatin architecture using a single dCas9 protein. FPs: fluorescent proteins; sgRNA: single-guide RNA.

This visual feature has made FPs a foundational component in the development of CRISPR-based *in vivo* imaging technologies. The core strategy involves directly fusing catalytically inactive CRISPR effector proteins such as dCas9 and dCas12 for DNA imaging and dCas13 for RNA imaging to FPs. When the dCas proteins are guided by sgRNA to target specific genomic loci or RNA molecules, the fused FPs emit signals upon laser excitation, enabling direct localization and real-time tracking of target nucleic acids under an optical microscope (**Figure 2A**)^7^, ^19^, ^21^. Compared to traditional FISH techniques, a significant advantage of the CRISPR-FP imaging system is that it does not require cell fixation or denaturation, thereby preserving cell viability^3^. This enables dynamic monitoring of target nucleic acid expression, localization, trafficking, and degradation in living cells and even *in vivo*, providing an unprecedented tool for studying biological processes such as gene regulation and RNA metabolism. Although the CRISPR-FP strategy has opened new avenues for genome visualization, early systems commonly faced challenges such as low signal-to-noise ratio (SNR), weak fluorescence intensity, and limited labeling efficiency at single genomic loci. To overcome these limitations, researchers have proposed various enhancement strategies aimed at increasing fluorescence signal strength, improving spatial resolution, and enhancing the ability to capture dynamic molecular processes. The SunTag system enhances signal amplification by fusing multiple tandem repeats of the Gcn4 peptide to the C-terminus of dCas9. Each Gcn4 motif can bind to a single-chain variable fragment antibody domain tagged with GFP, allowing multiple FPs to be recruited to a single dCas9 molecule. This “many-to-one” amplification strategy significantly improves the visualization of low-abundance DNA loci or rare transcripts^33^, ^34^. Building upon this, the MoonTag system introduces an orthogonal peptide-nanobody recognition pair relative to SunTag, effectively reducing nonspecific background while offering greater modularity and multiplexing capability. This allows for simultaneous tracking of multiple targets and multicolor labeling^79^. Both systems can be used in parallel within the same platform, providing CRISPR imaging with enhanced multidimensional signal readout capabilities. Another enhancement strategy is the split-FP system, in which a fluorescent protein is divided into two complementary fragments (GFP1-10 and GFP11), each fused to distinct molecular recognition elements (**Figure 2A**). Fluorescence is generated only when the two fragments come into close spatial proximity and reassemble into a complete fluorescent structure, thereby enabling dynamic visualization of molecular interactions^80^. Owing to its “switch-like” signal output, split-FP does not amplify fluorescence intensity, but significantly improves the SNR by minimizing background fluorescence. Therefore, it is not only considered a strategy to enhance imaging contrast but is also frequently regarded as a form of smart gated probe.

In addition to the direct fusion of FPs with Cas effectors, structural modification of sgRNA has emerged as a critical advancement in recent CRISPR/Cas imaging strategies (**Figure 2B**). By incorporating MS2 or PP7 into the sgRNA, researchers have engineered RNA-protein recognition modules that enable precise recruitment of FPs to target nucleic acid loci, thereby achieving high-specificity visualization of genomic and transcriptomic targets^20^, ^32^. Initially developed for single-locus tracking, this strategy evolved into the CRISPRainbow system, which integrates multiple recognition elements (e.g., MS2, PP7, and boxB) within the sgRNA and employs spectrally distinct fluorescent tags to enable simultaneous, multicolor imaging of multiple genomic loci in live cells^20^. To further enhance sgRNA stability and signal intensity, Ma *et al.* developed the CRISPR-Sirius system, in which an octet array of MS2 or PP7 aptamers is embedded within the four-way junction of the sgRNA. This configuration dramatically improves the recruitment efficiency of FPs and achieves an order-of-magnitude increase in signal intensity in human U2OS cells^81^. CRISPR-Sirius is currently considered the brightest and one of the most robust CRISPR-based imaging platforms, with its amplification capability able to boost signals by an order of magnitude, offering superior signal fidelity and spatiotemporal resolution even within complex chromatin environments.

In addition, structural optimizations of FPs themselves have provided crucial support for enhancing the performance of CRISPR imaging systems. Ultra-bright variants such as mNeonGreen^82^ and StayGold^83^ significantly increase signal intensity, while msGFP2, developed by Fernando M. Valbuena and colleagues, exhibits excellent folding stability and antioxidative properties, enabling stable fluorescence emission in complex intracellular environments and offering more reliable molecular tools for intracellular imaging^84^. Meanwhile, acid-resistant variants such as pHuji^85^ and Gamillus^86^ have expanded the applications of FPs within acidic organelles like lysosomes. Notably, the introduction of artificial intelligence is accelerating the functional evolution of FPs. Thomas Hayes and colleagues designed esmGFP based on the large language model ESM-3, which exhibits excellent brightness and folding ability, marking a breakthrough in AI-driven protein engineering within the field of FPs^87^.

In CRISPR/Cas imaging systems, FPs have evolved from simple fusion tags to precise tools with enhanced controllability, stability, and resolution. With advances in synthetic biology, protein engineering, and imaging algorithms, these probes are progressing toward greater intelligence, multimodality, and high-throughput capability, promising broader applications in dynamic epigenetic regulation, single-cell imaging, and real-time multi-omics studies.

### Fluorescent dye probes: high-brightness, deep-penetration targeted labeling tools

As a key tool in biological imaging, synthetic fluorescent dyes are increasingly becoming indispensable non-genetically encoded labeling agents in CRISPR live-cell imaging. Owing to their small molecular size, these dyes offer excellent optical properties and superior tissue penetration. Compared to genetically encoded FPs, synthetic dyes are smaller in size, exhibit higher quantum yields, possess broader excitation and emission spectra, and demonstrate outstanding photostability, making them well-suited for multicolor imaging, high-sensitivity detection, and long-term dynamic tracking. Representative molecules include cyanine dyes such as Cy3 and Cy5^88^, the Alexa Fluor series^89^, and the next-generation Janelia Fluor (JF) dyes^90^. Each class possesses distinct advantages and limitations: Cyanine dyes are valued for their synthetic accessibility and cost-effectiveness, serving as foundational tools in many assays; however, they can be susceptible to photobleaching and exhibit lower brightness compared to more advanced dyes^91^, ^92^. The Alexa Fluor series, engineered for enhanced performance, offer superior brightness, excellent photostability, and a wide range of well-characterized spectra, making them a reliable, versatile choice for multi-plexed imaging and quantitative applications, though their relatively large size and poor cell permeability can limit their use in live-cell imaging without invasive delivery methods. Notably, the JF dyes offer exceptionally high quantum yields, outstanding photobleaching resistance, and excellent cell membrane permeability, making them particularly advantageous for live-cell and deep-tissue imaging^93^. A key advantage of JF dyes is their environment-sensitive fluorescence, which mini-mizes background signal in aqueous environments and enables ultra-high-contrast imaging; their primary limitation lies in their more complex synthesis and higher cost.

Early applications of synthetic dyes in biological imaging were exemplified by FISH, which uses fluorescently labeled oligonucleotide probes to specifically detect target DNA or RNA sequences in fixed cells. Although highly specific, this technique requires cell fixation and denaturation, limiting its use in real-time or dynamic imaging^3^. To overcome the static limitations of FISH, CRISPR technology has been integrated into fluorescent probe design by covalently linking small-molecule fluorescent dyes to dCas family proteins, thereby enabling programmable live-cell imaging platforms. Currently, two main strategies are employed to combine synthetic dyes with the CRISPR system: (1) covalently anchoring fluorescent dyes to CRISPR-associated dCas effector proteins, primarily through self-labeling tag systems; and (2) engineering sgRNA structures by embedding RNA aptamers that specifically recruit fluorescent ligands, or by directly incorporating synthetic dyes into the sgRNA molecule to achieve precise labeling of target loci. Each approach has its own advantages, and together they have significantly advanced live-cell genome imaging technologies^8^.

**Figure 3:**
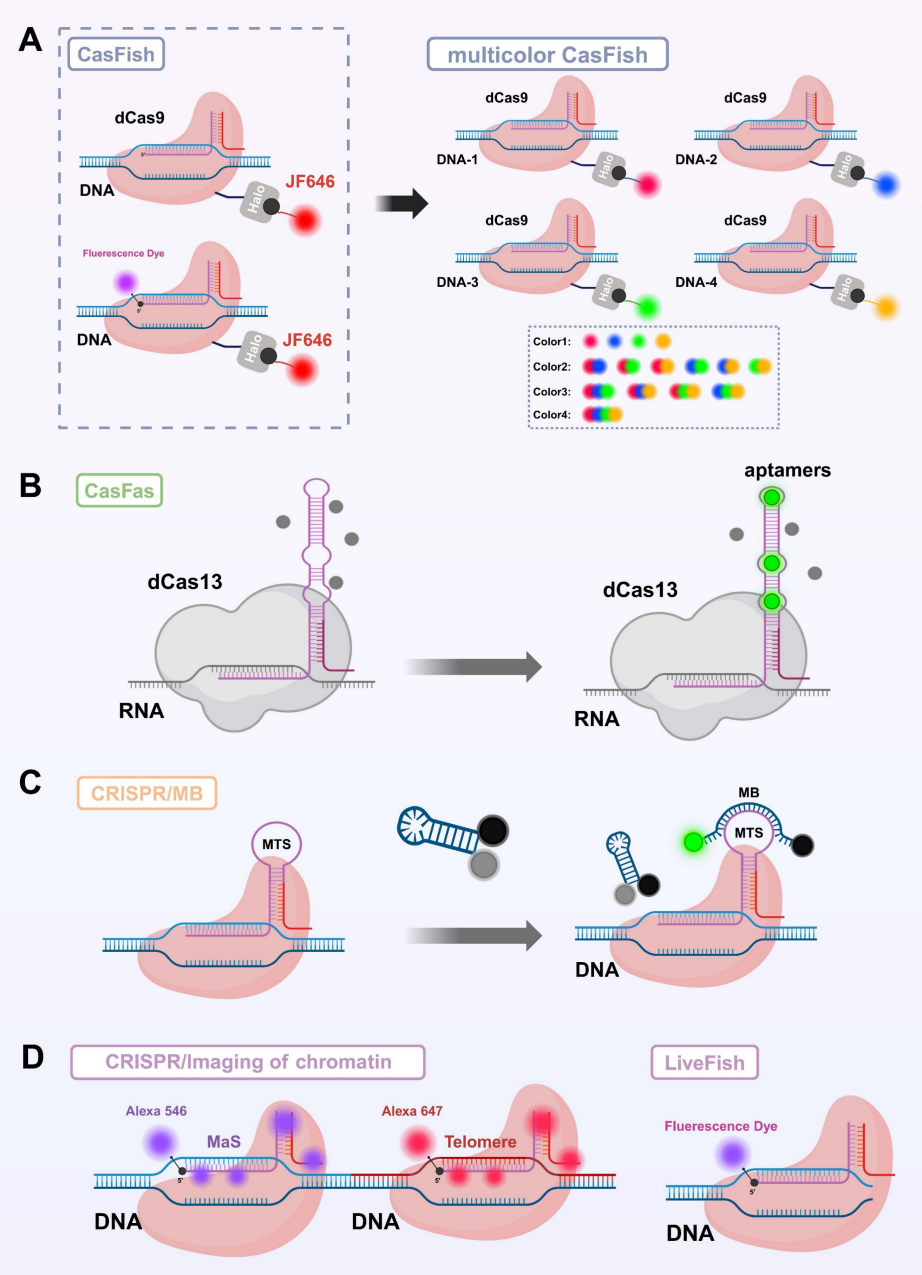
Synthetic fluorescent dye-based bioimaging strategies mediated by CRISPR/Cas systems. (A) HaloTag-Based Imaging System: The imaging system is constructed by fusing the HaloTag peptide (297 amino acids, 33 kDa), derived from bacterial haloalkane dehalogenase, to the C-terminus of dCas9 to form a dCas9-HaloTag fusion protein. This fusion protein covalently binds to organic fluorescent dyes (Halo ligands), enabling fluorescence emission. This strategy is also applied in the CASFISH method, where fluorescently labeled sgRNA is combined with the fusion protein for FISH imaging of genomic loci. (B) CRISPR/MB System: By integrating the molecular beacon (MB) target sequence (MTS) into the tetraloop structure of the sgRNA, the MB hybridizes with the MTS, leading to the opening of its hairpin structure. This conformational change separates the fluorophore from the quencher, thereby activating a fluorescent signal. This strategy enables controllable fluorescence imaging without the need for fusion proteins. (C) RNA Aptamer-Based Imaging System (CasFAS): The RNA aptamer “Broccoli” is inserted into the sgRNA to form sgRNA-2xBroccoli. Upon binding with the small-molecule fluorescent probe DFHBI, fluorescence is activated, enabling imaging at the level of transcribed RNA. (D) Fluorescent Oligonucleotide Labeling Strategy: This approach includes modifying sgRNA with multiple fluorophores to enhance imaging signals, as well as the LiveFISH method, which involves the co-delivery of fluorescently labeled sgRNA and dCas9 to form a fluorescent ribonucleoprotein complex for real-time visualization of target sites in living cells. FISH: fluorescent *in situ* hybridization; MB: molecular beacon; MTS: molecular beacon target sequence.

Self-labeling protein tag systems associated with Cas effector anchoring primarily include three widely used strategies: HaloTag, SNAP-tag, and CLIP-tag^94^. These systems enable the precise covalent attachment of fluorescent dyes to dCas proteins through highly specific reactions, achieving live-cell imaging with a high SNR and low background. Among them, HaloTag is an engineered derivative of a dehalogenase enzyme that forms a stable covalent bond with fluorescent substrates containing a halogenated alkane structure under neutral conditions^95^. In CRISPR applications, researchers typically fuse HaloTag to the C-terminus of dCas9 (or dCas13) and achieve efficient labeling by introducing modified fluorescent dyes into the cells. Based on this strategy, Deng and colleagues developed the CASFISH system^96^, which employs a dCas9-HaloTag fusion protein combined with the JF646 fluorescent dye to achieve precise visualization of various genomic regions in mouse embryonic fibroblasts and HeLa cells (**Figure 3A**). These regions include repetitive sequences such as pericentromeric heterochromatin and G-rich telomeric repeats, as well as non-repetitive genomic loci. This system provides a gentle, flexible, and high signal-to-noise imaging method for high-resolution analysis of genome spatial organization and chromatin states in fixed cells; SNAP-tag is an engineered tag derived from O⁶-alkylguanine-DNA alkyltransferase that can rapidly form covalent bonds with fluorescent probes modified with benzyl-guanine (BG) groups^97^. Similar to HaloTag, SNAP-tag is often fused to dCas proteins for imaging targeted DNA or RNA. Compared to HaloTag, SNAP-tag has a smaller molecular size and better solubility, making it more suitable for precise labeling within the nuclear or cytoplasmic environment. However, because SNAP-tag is derived from the human DNA repair enzyme AGT, it can exhibit higher background labeling due to recognition of the BG substrate by endogenous AGT. In contrast, HaloTag, which is derived from a bacterial protein, does not have this issue, although it is slightly larger in size^98^; CLIP-tag is an engineered mutant of SNAP-tag that specifically recognizes fluorescent probes modified with benzyl-cytosine (BC)^99^. Due to the excellent orthogonality in substrate recognition between SNAP-tag and CLIP-tag, both can be used together within the same system to achieve multicolor imaging and simultaneous tracking of multiple targets.

Compared to fusion tagging or chemical modification of Cas proteins, engineering modifications of sgRNA have gradually become a key approach for constructing high-performance imaging probes due to sgRNA's small molecular size, structural flexibility, mature synthesis methods, and minimal impact on CRISPR complex activity^8^. Currently, sgRNA-based fluorescent dye labeling strategies mainly fall into two categories: insertion of fluorescent recruitment elements into sgRNA and direct chemical modification of sgRNA^8^, ^100^. In the development of engineered fluorescent labeling strategies for sgRNA, the MS2 system was one of the earliest and most widely used methods. This approach inserts MS2 RNA tag sequences into the sgRNA and employs MS2 coat proteins (MCP) fused with FPs to achieve indirect labeling of the target RNA^32^. The MS2 system performs excellently in multiplex imaging and studies of RNA-protein complex dynamics; however, its implementation is relatively complex, requiring co-expression of multiple components, and the signal-to-noise ratio is often compromised by nonspecific background interference. Subsequently, RNA aptamer insertion strategies were proposed and have been widely adopted (**Figure 3C**)^22^, ^101^. Spinach and Mango RNA aptamers can specifically bind distinct small-molecule fluorescent dyes, with Spinach recognizing 3,5-difluoro-4-hydroxybenzylidene imidazolinone (DFHBI), whereas Mango binds thiazole-orange derivatives such as TO1-Biotin^102^, ^103^. These dyes are quenched in their free state and become fluorescently activated only upon binding to the aptamers, resulting in a high SNR. By inserting multiple copies of the aptamers, the fluorescence signal can be effectively amplified, making them well-suited for real-time dynamic imaging with high spatial and temporal resolution^104^. However, the insertion of aptamers requires substantial modification of the sgRNA structure, which may compromise its stability, reduce its binding efficiency with dCas proteins, and interfere with intracellular transport, thereby limiting its general applicability in live-cell imaging^105^. To address the structural interference caused by aptamer insertion, the CRISPR/MB system was developed. This system integrates a molecular beacon (MB) target sequence (MTS) into the tetraloop structure of the sgRNA, allowing the MB probe to specifically hybridize with the MTS (**Figure 3B**). Upon binding, the hairpin structure of the MB is opened, separating the fluorophore from the quencher and releasing a fluorescent signal, thereby enabling high SNR imaging^106^. Although this approach improves signal quality, it still relies on the efficient delivery of exogenous probes, adding to the operational complexity.

In recent years, to simplify imaging workflows and enhance signal stability, researchers have proposed a strategy of covalently labeling sgRNA with fluorescent dyes via chemical modification. Originating from techniques widely used in protein labeling^107^, this approach has been extensively applied in high-resolution imaging, while its adaptation to RNA remains under active investigation. By directly attaching synthetic fluorescent dyes to the 5' or 3' end of sgRNA, this method offers a simple, broadly applicable labeling strategy with minimal structural interference (**Figure 3D**)^108^, ^109^. Among these approaches, 5' end modification is widely adopted due to its high synthetic efficiency and good functional compatibility. Compared to aptamer-ligand systems, this covalent labeling strategy offers superior signal stability and avoids signal variability caused by fluctuations in binding efficiency, making it particularly suitable for high-throughput screening and other applications requiring high signal consistency^109^. Wang *et al.* developed the LiveFISH system by labeling the 5' end of sgRNA with fluorescent dyes and co-delivering it with dCas9 protein, enabling simultaneous visualization of multiple chromosomal repetitive regions in mammalian cells^110^. To further expand its applicability to non-repetitive loci and studies of chromatin dynamics, Zhu *et al.* subsequently optimized the system by designing the Oligo-LiveFISH strategy^111^, which combines dCas9 with synthetic oligonucleotide probes to achieve precise labeling of complex or low-copy genomic regions. By optimizing the design of the gRNA pool, this technique achieved super-resolution dynamic tracking of endogenous non-repetitive gene loci, with a spatial resolution of 20 nm and a temporal resolution of 50 ms. Although the aforementioned strategies show promising potential for *in vivo* imaging, they still face several challenges. The variety of fluorescent dyes and labeling sites remains limited, and the functional integrity of modified sgRNA as well as its binding efficiency with dCas9 require further optimization. Additionally, the photostability and biocompatibility of some organic dyes within cells need to be improved^8^.

### Smart gated fluorescent probes: dynamic regulation and high spatiotemporal resolution imaging

In recent years, to meet the growing demand for precise visualization of biomolecules across complex spatial and temporal dimensions, smart gated fluorescent probes have emerged. These probes not only maintain the high brightness and specificity required for conventional imaging but also possess input-responsive activation capabilities, meaning their fluorescence is triggered only under specific conditions and enables switch-like signal control. This design significantly reduces background noise, enhances imaging contrast, and offers distinct advantages in advanced applications such as single-cell analysis, multimodal imaging, and real-time dynamic visualization (**Table 3**).

#### Photoactivatable gated probes: enabling non-invasive control with high spatiotemporal resolution

Photoactivatable gated probes are among the earliest intelligent strategies applied in CRISPR live-cell imaging. By using light to trigger fluorescence activation, they enable high spatiotemporal resolution imaging, making them well-suited for dynamic tracking and precise localization.

**Figure 4:**
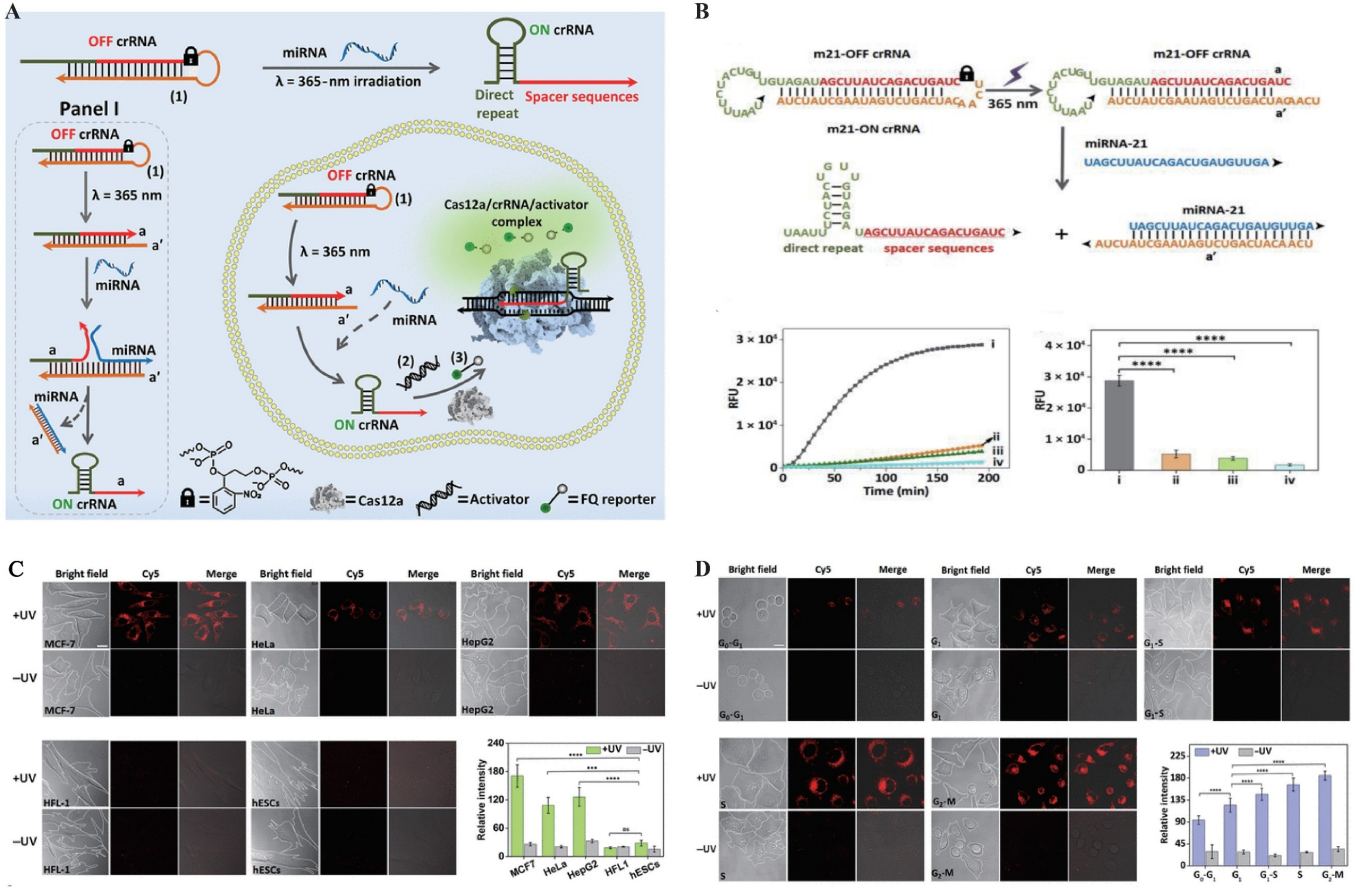
Photoactivatable Gated Probes. (A) Schematic illustration of the light-controlled reaction module coupled with the CRISPR-Cas12a (LAC12a) mechanism for specific recognition and signal amplification of miRNA-21. (B) Time-dependent fluorescence intensity of the LAC12a system under different conditions: (i) with miRNA-21 (10⁻⁷ M) and UV activation; (ii) without miRNA-21 but with UV activation; (iii) with miRNA-21 (10⁻⁷ M) but without UV activation; and (iv) without miRNA-21 and without UV activation. RFU, relative fluorescence units. (C) Confocal microscopy images (bright-field and fluorescence) and quantified fluorescence intensities showing the performance of the LAC12a sensing system in different cell lines (MCF-7, HeLa, HepG2, HFL-1, and hESC) under photoactivated (+UV) and non-photoactivated (-UV) conditions, with the strongest response observed in MCF-7 cells. Statistical analysis revealed significant differences (****P < 0.0001, ns, not significant). (D) Confocal microscopy images and quantified fluorescence intensities of MCF-7 cells synchronized at different cell-cycle phases (G0/G1, G1, G1/S, S, and G2-M), demonstrating the applicability of the LAC12a mechanism for miRNA-21 detection during dynamic cell-cycle progression. Adapted with permission from^115^. Copyright 2024, Science.

In the early development of photoactivatable gated probes, irreversible photocaged groups were widely employed as a key strategy for enabling on-demand activation of fluorescence signals. These photosensitive moieties undergo photolytic cleavage upon exposure to specific wavelengths of light, thereby releasing the caged functional sites and allowing precise control over fluorescence emission^112^. Common photocaged groups include o-nitrobenzyl (NB)^113^ and 4,5-dimethoxy-2-nitrobenzyl (DMNB)^114^, which are widely used for precise regulation of nucleic acids and proteins due to their high photo-reactivity and well-established synthetic routes. In CRISPR live-cell imaging, these photocaged groups block critical binding sites on Cas proteins or sgRNA, keeping the CRISPR/Cas system in an “inactive” state under dark conditions, thereby effectively suppressing background fluorescence caused by non-specific binding^115^, ^116^. Hemphill *et al.* introduced a photocaged lysine at the K866 site of Cas9 protein, enabling reversible light control of Cas9 nuclease activity^117^. Liu *et al.* developed a blue light-activated crRNA gating strategy by incorporating a NB photocaged group into the hairpin structure of crRNA (**Figure 4**), which releases active crRNA only upon blue light irradiation, thereby activating Cas12a's trans-cleavage function and successfully achieving spatiotemporally specific imaging of target miRNA^115^. Similar caging strategies have also been widely applied to DNAzymes. As structurally stable and easily engineered nucleic acid enzymes, DNAzymes possess excellent photoactivatable properties, significantly expanding the potential of light-controlled probes for dynamic monitoring in live cells and even *in vivo*, thereby advancing the fields of precision medicine and molecular diagnostics. By introducing photocaged groups into key nucleotides or catalytic sites of DNAzymes, researchers can achieve precise “light switch” control over their enzymatic activity, enabling spatiotemporal regulation of function^118^, ^119^. Furthermore, by integrating DNAzymes with the Cas9 system, molecular logic gates have been constructed to visualize the dynamic spatiotemporal distribution of metal ions in living organisms^119^.

However, irreversible light-controlled systems have certain limitations: once activated, they cannot be switched off, making them unsuitable for repeated imaging applications. To address this, researchers have explored reversible light-controlled systems based on protein interactions. A representative example is the LACE system developed by Polstein *et al.*, which utilizes the light-responsive proteins CRY2 and CIB1. Upon light blue light (approximately 450-488 nm) illumination, the light-responsive protein pair CRY2 and CIB1 undergo dimerization, facilitating the precise recruitment of the transcriptional activator VP64 to specific genomic loci. This enables optogenetic control of dCas9-mediated transcriptional regulation^120^. However, traditional photocaged groups typically rely on ultraviolet (UV) light activation, which suffers from limited tissue penetration and high phototoxicity^121^. To address these challenges, recent studies have explored the integration of CRISPR/Cas systems with near-infrared (NIR) responsive materials, employing upconversion nanoparticles (UCNPs) to convert NIR light into UV light, thereby enabling remote activation of CRISPR/Cas activity in deep tissues^122^, ^123^. Building on this strategy, Liu *et al.* developed a NIR-activated UCNPs-CRISPR/Cas12a fluorescent biosensing system for the specific detection of tumor-related mRNA *in vivo*^48^. In this system, crRNA modified with a photocleavable PClinker group was electrostatically adsorbed onto the surface of UCNPs. Upon irradiation with 808 nm NIR light, the UCNPs emitted UV light that efficiently removed the protective group, releasing active crRNA. Additionally, MnO₂ nanosheets were incorporated to enhance cellular uptake and release Mn²⁺ intracellularly, thereby boosting Cas12a catalytic activity. This enabled an integrated strategy of light-controlled activation, target recognition, and fluorescence imaging^48^. Subsequently, Sun *et al.* further improved the system's sensitivity by introducing a hybridization chain reaction (HCR) amplification module, allowing robust signal amplification and reliable imaging even under conditions of low miRNA expression^124^.

In summary, light-gated probes based on irreversible photocleavable groups have provided an early and effective strategy for spatiotemporal control in CRISPR live-cell imaging. Their development laid the foundation for current intelligent CRISPR probe systems and catalyzed the emergence of advanced approaches, such as reversible photoactivation and NIR-triggered regulation via UCNPs materials.

#### Nucleic acid-responsive gated probes: precisely regulating CRISPR/Cas system activity through nucleic acid conformational changes

Nucleic acid-responsive gated probes represent an important advancement in the intelligent regulation strategies of the CRISPR/Cas system, fully leveraging the high specificity of nucleic acid sequence recognition and the dynamic modulation capability of conformational changes. This approach introduces elements such as aptamers and hairpin structures to construct probes with “locking-response-unlocking” functions, enabling precise spatial recognition and structural activation. Without relying on external physical stimuli, these probes exhibit good biocompatibility and high imaging specificity, showing great potential for multidimensional imaging and target detection within cells.

In nucleic acid-responsive pathways, aptamers are widely employed in the construction of intelligent CRISPR-based sensors due to their high-affinity recognition of intracellular functional molecules such as ATP, ADP, and VEGF. As ssDNA or RNA sequences obtained through artificial selection, aptamers undergo structural unlocking or conformational changes upon binding to their target molecules^125^, ^126^. In recent years, researchers have embedded aptamers into the target strands of CRISPR/Cas systems, enabling conformational switching of the aptamer in response to specific target molecules. This structural change modulates the accessibility of the target strand, thereby regulating the activation of the CRISPR/Cas system^127^-^130^. This strategy significantly enhances the specificity and controllability of molecular-responsive probes. Pan *et al.*^128^ applied this approach to design and develop an ATP-responsive CRISPR-Cas12a nano-sensor, in which the aptamer undergoes a structural rearrangement upon ATP binding, releasing a pre-hybridized activator strand to trigger Cas12a trans-cleavage. This design allowed sensitive and selective ATP detection under physiological conditions, demonstrating the feasibility of integrating aptamer-CRISPR modules for *in situ* molecular imaging.

Hairpin structures, as another representative form of nucleic acid secondary structures, also play a key role in the gate regulation of CRISPR/Cas systems. Their reversible open-close behavior allows the exposure of critical recognition sequences under specific conditions, enabling the construction of structured probes with a “lock-response-unlock” mechanism. In the absence of target sequences, the hairpin remains in a self-locked state to suppress CRISPR/Cas activation; upon recognition of specific nucleic acid targets, the structure unfolds, thereby triggering the CRISPR/Cas function^131^, ^132^. However, similar to aptamers, hairpin-based systems also face challenges in complex physiological environments, such as high background noise and limited tissue penetration. Further optimization is needed to enhance their performance in *in vivo* imaging applications.

In summary, nucleic acid-responsive gate probes leverage the high specificity of nucleic acid recognition and structural tunability to achieve intelligent self-locking and precise activation of the CRISPR/Cas system. These probes demonstrate excellent specificity and good biocompatibility in cellular imaging and target detection.

#### Logic gate probes: precise regulation strategies based on multiplex signal cooperative recognition

The concept of "molecular logic gates" was first proposed by de Silva and colleagues in 1993. They synthesized an "AND"-type fluorescent probe that only produced a fluorescent response in the simultaneous presence of H⁺ and Na⁺^133^, laying the foundation for subsequent intelligent molecular recognition systems based on chemical and biological signal inputs. Building on this foundation, logic gate probes have been developed that integrate multiple biological signals and leverage the programmability and dynamic assembly capabilities of nucleic acid structures to achieve precise regulation of the CRISPR/Cas system. These probes represent an advanced form of intelligent gating strategies. Beyond recognizing single signals, they can perform Boolean logic operations such as AND, OR, and NOT to enable cooperative multi-signal recognition and precise activation, effectively reducing false triggers and enhancing the specificity and robustness of the system^134^, ^135^.

**Figure 5:**
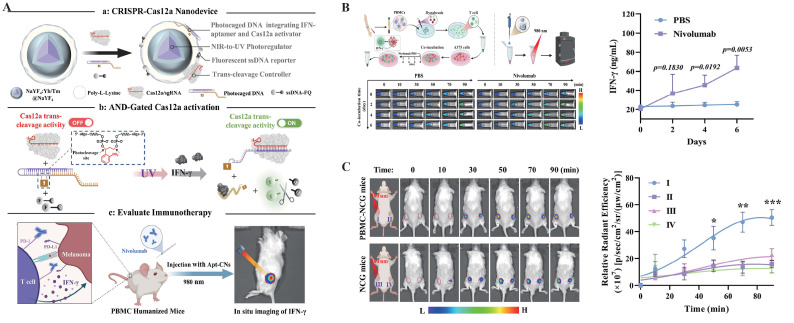
Logic Gate Probes. (A) Schematic illustration of the design principle of near-infrared light-activated aptamer-CRISPR nanodevices (Apt-CNs). The system employs an “AND” logic gate strategy to enable *in situ* real-time monitoring of IFN-γ release during immune checkpoint blockade therapy in humanized mice. (B) In a co-culture system of A375 cells and human T cells activated with anti-CD3/CD28 Dynabeads, Apt-CNs were used for *in situ* fluorescence monitoring of IFN-γ release. The cultures were treated with PBS as a control or with nivolumab (10 μg/mL) to block PD-1/PD-L1 and activate T cells. Fluorescence images were acquired at different time points under 980 nm laser irradiation (2 min, 250 mW cm⁻²). The quantified fluorescence signals reflect the dynamic changes of IFN-γ over time, which were further validated by ELISA. Data are presented as mean ± SD (n = 3), and statistical significance was assessed by two-sided t-test. (C) Fluorescence images of NCG and PBMC-NCG mice bearing bilateral A375 tumors after intra-tumoral injection of Apt-CNs, with tumors indicated by red circles. Fluorescence signals at tumor sites were quantified over time and plotted as curves (mean ± SD, n = 3). Adapted with permission from^14^, copyright 2024 American Chemical Society.

Currently, the design of logic gate probes integrated with the CRISPR system primarily relies on the combination of multiple nucleic acid elements, with aptamer systems and hairpin structures being typical examples. These elements construct hierarchical gating networks through complementary base pairing or conformational rearrangement^136^-^139^. Among various logic modes, the AND gate strategy demonstrates greater maturity and stability. Compared to OR and NOT logic, the AND gate activates the CRISPR system only when multiple input signals are simultaneously present, significantly enhancing specificity and safety. This feature makes it especially suitable for applications such as *in vivo* imaging and targeted therapy, where tolerance for “false activation” is extremely low. Based on this, Liu *et al.* innovatively integrated UCNPs, NIR light, and the aptamer system to achieve photo-regulation of aptamer conformation (**Figure 5**). This strategy successfully enabled high spatiotemporal resolution imaging of IFN-γ in Tumor-T cell co-culture systems and multiple mouse models, significantly reducing background signals and allowing for deep tissue imaging^14^.

### Nanomaterial-based probes: achieving multifunctional integration and precise delivery through nanomaterials

**Figure 6:**
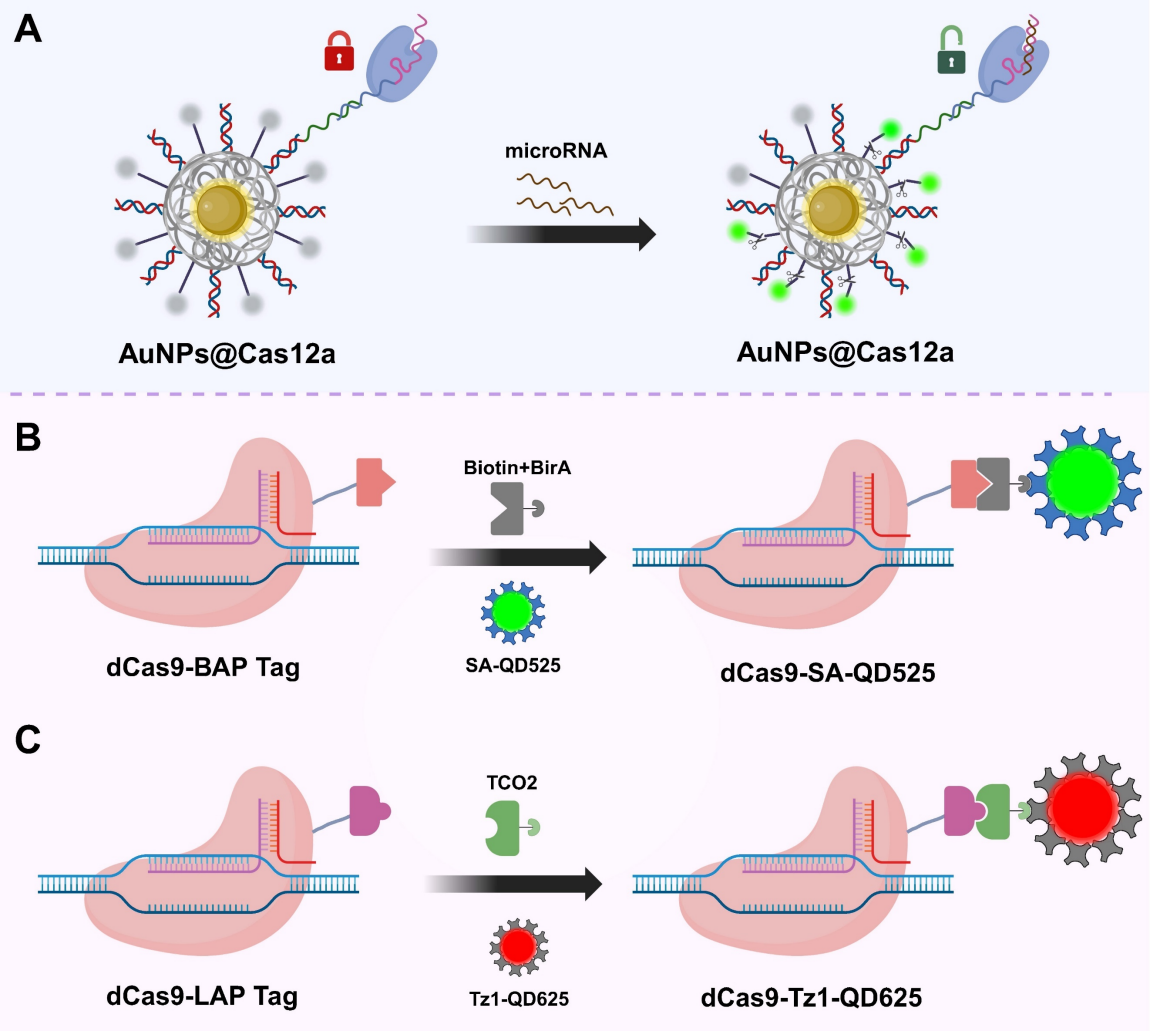
Schematic illustration of CRISPR/Cas-based bioimaging using nanoprobes. (A) AuNPs for Cas system delivery^49^. The surface of AuNPs can be densely functionalized with thiol-modified oligonucleotides containing disulfide bonds, enabling multivalent anchoring of sgRNAs or fluorescent probes while efficiently quenching fluorescence signals. These features allow AuNPs to serve as a “switch” for the loading and activation of the Cas12a system; (B) QD-labeled CRISPR system based on LplA(8). In this method, dCas9 is fused with a lipoic acid acceptor peptide (LAP), which is ligated with TCO2 in the presence of LplA. Tz1-QD are subsequently conjugated to TCO2, forming dCas9-labeled tetrazine-QD complexes for visualizing target DNA; (C) Quantum dot labeling strategy based on the biotin-streptavidin system. In this approach, dCas9 is fused with a BAP-tag and biotinylated by the biotin ligase BirA. SA-QD are then bound to the biotinylated dCas9, enabling fluorescent labeling and visualization of target DNA. AuNPs: gold nanoparticles; SA-QD: streptavidin-conjugated quantum dots; QDs: quantum dots; LplA: lipoic acid ligase; BAP-tag: biotin acceptor peptide; TCO2: trans-cyclooctene; Tz1-QD: tetrazine-modified quantum dots.

As a vital complement to *in vivo* tumor imaging technologies^140^, nanomaterial-based probes have shown great potential in enhancing signal intensity, improving delivery efficiency, and enabling multifunctional integration, owing to their unique size effects, surface modifiability, and excellent biocompatibility^141^. Currently, research integrating CRISPR/Cas systems for *in vivo* imaging primarily focuses on two types of nanomaterials: gold nanoparticles (AuNPs) and quantum dots (QDs) (**Figure 6**).

AuNPs, due to their excellent photothermal responsiveness and strong affinity for nucleic acids, have demonstrated remarkable performance in CRISPR-related controlled release platforms^142^. Their surfaces can be densely functionalized with thiol-modified oligonucleotides containing disulfide bonds, enabling multivalent anchoring of sgRNAs or fluorescent probes while efficiently quenching fluorescence signals^143^. With technological advancements, researchers have further developed DNA and RNA modified AuNPs synthesized via rapid microwave-assisted methods, significantly improving the efficiency and structural stability of probe construction^144^. Moreover, this multivalent loading strategy significantly increases the local concentration of sgRNAs and probes while enriching Mg²⁺ ions essential for CRISPR/Cas system activity, thereby enhancing both system activation and imaging signal intensity. Additionally, AuNPs exhibit excellent cellular uptake capabilities, effectively facilitating transmembrane delivery. Leveraging these properties, Yuan *et al.* combined AuNPs with Cas12a to achieve sensitive and real-time imaging of specific microRNAs in living cells, demonstrating the promising potential of CRISPR/Cas-based nanorobots for functional monitoring and regulation in live-cell environments (**Figure 6A**)^49^.

QDs are a class of luminescent semiconductor nanoparticles ranging in size from 1 to 20 nm. Compared to traditional organic fluorescent dyes and FPs, QDs exhibit higher brightness, narrower emission spectra, and outstanding photostability, making them ideal fluorescent probes for high-sensitivity imaging applications^145^. In CRISPR/Cas systems, QDs are commonly covalently linked to Cas proteins via ligase-mediated conjugation using lipoic acid ligase or biotin-streptavidin interactions, thereby enabling targeted recognition and fluorescent imaging capabilities (**Figure 6B-C**)^146^, ^147^. Based on this, Ma *et al.* developed a dual-color CRISPR/Cas imaging system by integrating QD-labeled dCas9 and sgRNA to visualize HIV proviral DNA within host cells^147^. However, despite their excellent optical properties, the application of QDs in CRISPR/Cas live-cell imaging still faces certain limitations. After entering cells, QDs tend to be trapped in lysosomes and are prone to forming fluorescent aggregates in the high-concentration intracellular environment, which adversely affects their imaging performance and stability^146^. These challenges urgently require solutions through surface modification optimization and delivery pathway design to further advance the practical application of QDs in *in vivo* imaging.

### Multimodal probes: integrated CRISPR/Cas platforms combining multiple imaging and therapeutic functions

With the ongoing advancement of CRISPR/Cas technology in molecular diagnostics and precision therapy, single-mode imaging approaches are increasingly inadequate for addressing the complex needs of various disease states^148^. In response, multimodal probes that combine multiple imaging signals with therapeutic functions have emerged as a critical direction in the development of CRISPR platforms. These probes not only provide more comprehensive diagnostic information and enhance diagnostic accuracy, but also enable integrated theranostic strategies, particularly advantageous in diseases such as cancer, which are characterized by high heterogeneity and complex spatial progression (**Table 4**)^149^.

**Figure 7:**
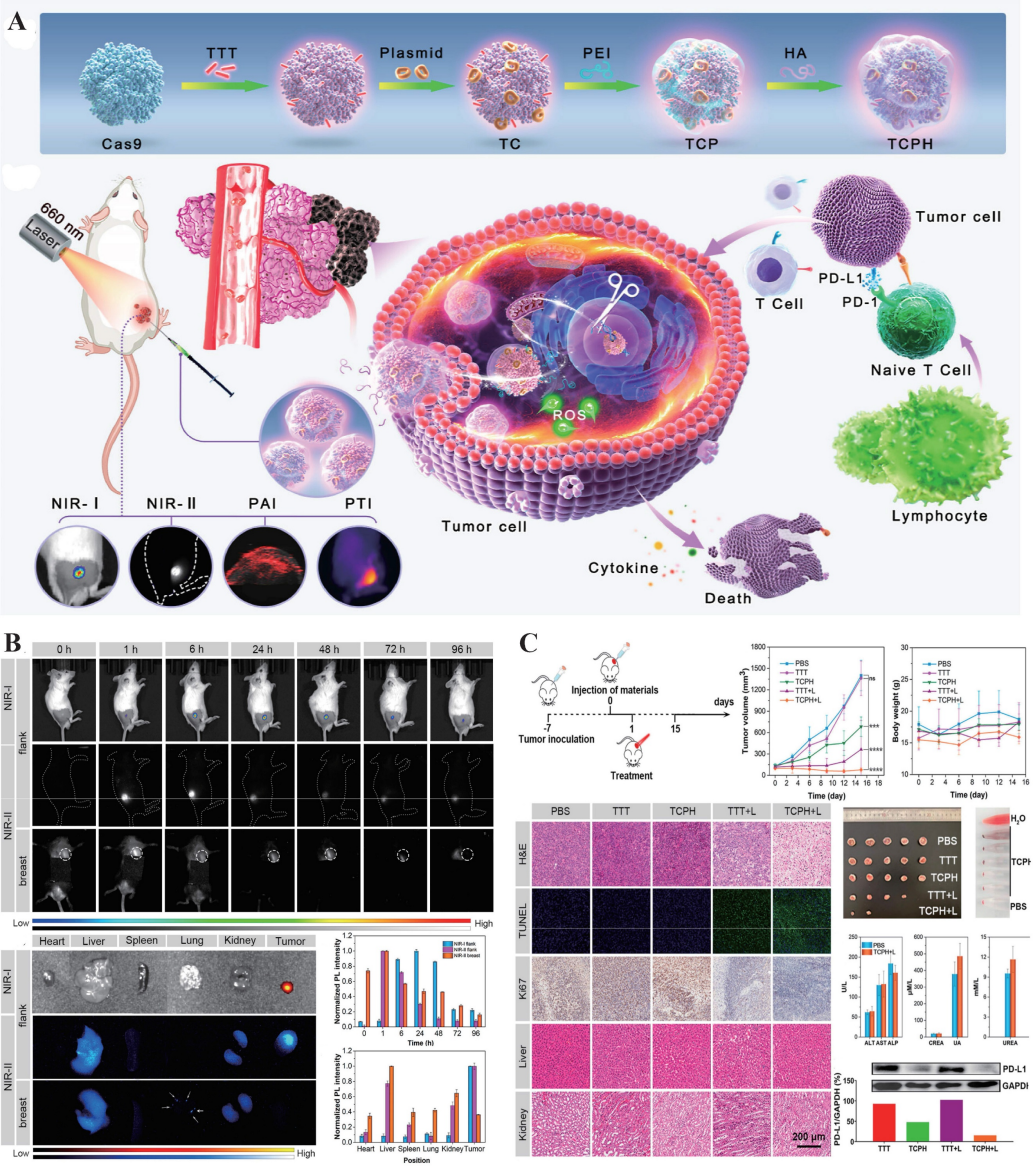
Multimodal Probes. (A) Schematic illustration of the construction of TCPH nanoparticles (NPs) using a stepwise assembly strategy. (B) *In vivo* and *ex vivo* NIR-I/NIR-II fluorescence imaging (FLI) of 4T1 tumor-bearing BALB/c mice at different time points after intratumoral injection of TCPH, showing tumors and major organs. The corresponding normalized photoluminescence (PL) intensities of tumors and organs are also presented. (C) Evaluation of the antitumor efficacy of TCPH in a unilateral subcutaneous 4T1 tumor model, including the *in vivo* PDT/PTT/immunotherapy procedure, tumor growth and body weight monitoring, histological analyses of tumor and major organs (H&E, TUNEL, Ki67), tumor resection, hemolysis test, hepatic and renal function assays, and representative western blot (WB) patterns. Adapted with permission from^10^, copyright 2024 American Chemical Society.

Currently, the development of multimodal CRISPR/Cas platforms for *in vivo* imaging and therapy primarily centers around two classes of enzymes: Cas9 and Cas13. Cas9, known for its ability to induce targeted double-strand breaks in DNA, has been widely applied in the field of gene therapy^28^. Researchers have combined Cas9 with photosensitizers to construct nanomachines that integrate gene editing and photodynamic therapy, enabling precise intervention at tumor sites and real-time visualization of the therapeutic process. For example, Wang *et al.* developed a multifunctional nanoplatform, TCPH, by integrating Cas9 with an AIE photosensitizer and a polyethyleneimine/hyaluronic acid coating (**Figure 7**). This platform not only enables gene intervention but also induces synergistic photodynamic and immune-mediated killing of tumor cells, demonstrating significant potential in cancer therapy^10^. In contrast, Cas13, with its RNA-targeting and trans-cleavage capabilities shows greater promise in live-cell imaging^51^. Related multimodal studies often rely on NIR light-controlled systems, combining fluorescence or UCNPs with photodynamic therapy to construct high spatiotemporal resolution theranostic platforms for deep tissue applications, thereby advancing the practical implementation of CRISPR systems in *in vivo* multimodal imaging and precision intervention^150^.

Moreover, although *in vivo* applications are still in their early stages, the construction of multimodal CRISPR probes in the field of *in vitro* diagnostics has already demonstrated higher complexity and integration. In particular, Cas12a-based multimodal systems have been developed by coupling the enzyme with various output modules such as colorimetric, fluorescent, and surface-enhanced Raman scattering (SERS) signals, creating multi-signal cross-verification platforms that significantly enhance detection sensitivity and anti-interference capability^151^, ^152^. In the future, with continued advances in nanomaterials, interface engineering, and enzymatic reaction regulation, such *in vitro* multimodal CRISPR systems are expected to be extended to *in vivo* imaging and *in situ* theranostics, offering powerful tools for precise intervention in complex disease conditions.

In summary, multimodal CRISPR/Cas probes offer new avenues for early disease screening, dynamic monitoring, and personalized therapy. With ongoing advancements in nanomaterials and bioresponsive mechanisms, these platforms are expected to play a pivotal role in the future of precision medicine.

## CRISPR/Cas *In Vivo* Delivery Systems: From Vector Construction to Targeted Transport

**Figure 8:**
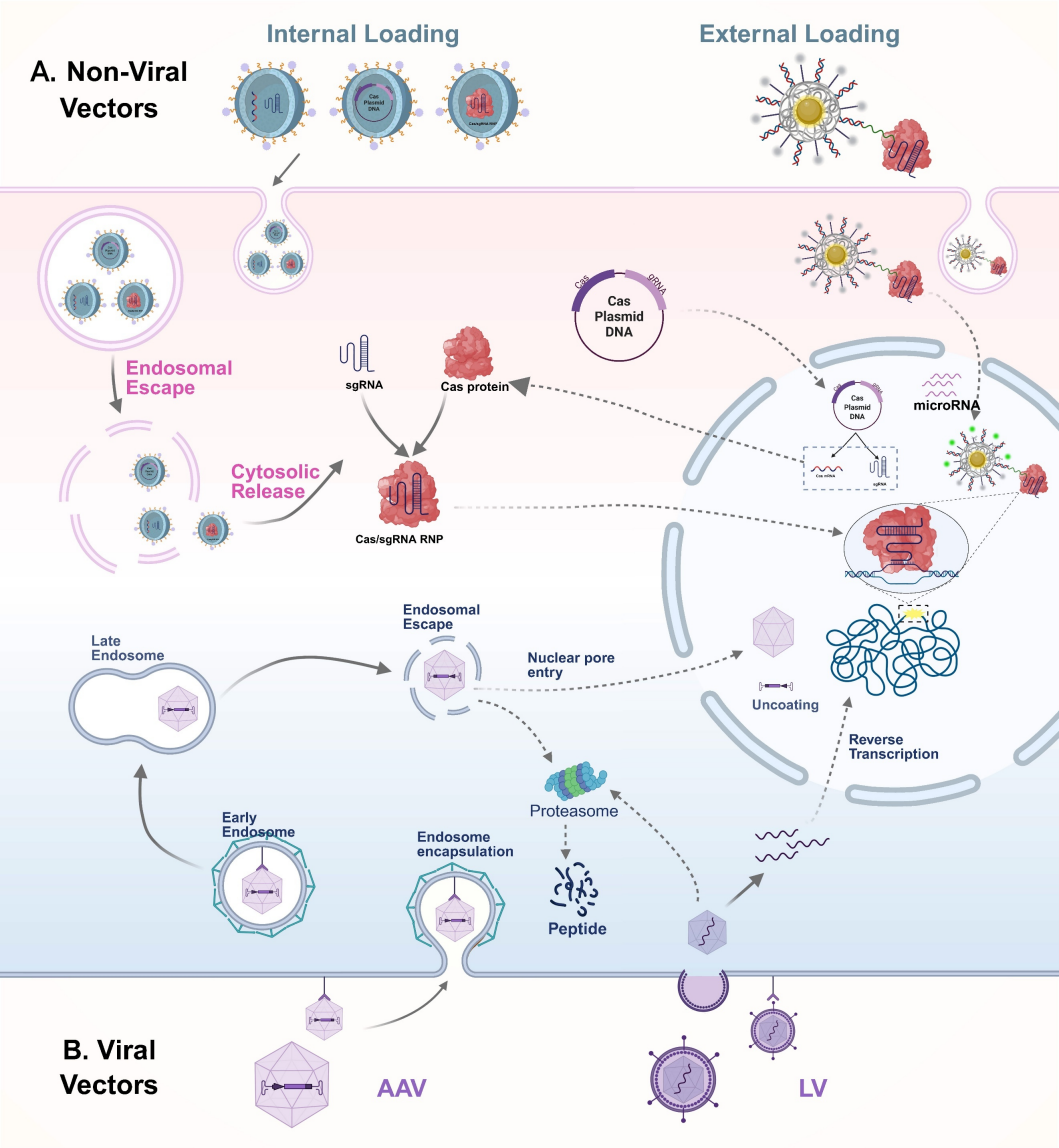
For *in vivo* delivery of the CRISPR/Cas system, both viral and non-viral vectors are commonly employed, each with distinct mechanisms and application advantages. Among viral vectors (B), AAV and LV are the most widely used. Both bind to specific receptors on the surface of target cells to initiate infection and are subsequently internalized. After cellular entry, AAV is capable of escaping from endosomes and traversing the nuclear membrane to enter the nucleus, although proteasome-mediated capsid degradation may also occur in the cytoplasm. In contrast, lentiviruses fuse directly with the cell membrane to release their RNA genome into the cytoplasm, where reverse transcription occurs to generate double-stranded complementary DNA, which is then integrated into the host genome, enabling long-term expression of the Cas system. Compared with viral vectors, non-viral delivery systems offer enhanced biosafety and greater versatility in cargo formats, including plasmid DNA, mRNA, and pre-assembled RNPs (A). These systems can be designed for either internal or external cargo loading. Typically, non-viral vectors enter cells via endocytosis and must escape from endosomes to release their cargo into the cytoplasm. Depending on the delivery form, plasmid DNA needs to reach the nucleus for transcription, mRNA is translated in the cytoplasm to produce Cas proteins, and RNPs can directly translocate into the nucleus to target specific DNA sequences. AAV: adeno-associated virus; CRISPR: clustered regularly interspaced short palindromic repeats; Cas: CRISPR-associated protein; RNP: ribonucleoprotein complexes; LV: lentivirus.

In recent years, with the widespread application of CRISPR/Cas systems in *in vivo* imaging and therapy, the efficient and safe delivery of these systems to target tissues and cells has become a central research focus. Currently, delivery strategies are broadly categorized into viral and non-viral vectors, with their transport mechanisms illustrated in **Figure 8**. Viral vectors offer high transduction efficiency and stable gene expression, making them suitable for various *in vivo* gene editing applications. However, their clinical translation is limited by issues such as immunogenicity, restricted cargo capacity, and potential risks of genomic integration. In contrast, non-viral systems, owing to their excellent biocompatibility, structural programmability, and low immunogenicity, have shown great promise in CRISPR delivery, particularly in multimodal imaging and image-guided therapy. A comparative summary of the advantages and representative applications of these delivery systems is presented in **Table 5**.

### Viral delivery system

Viral vectors are the mainstream tools for achieving efficient *in vivo* delivery of the CRISPR/Cas system, primarily including adeno-associated virus (AAV), lentivirus (LV), and adenovirus (AdV). Among these, AAV has become the preferred platform for *in vivo* CRISPR/Cas delivery due to its low immunogenicity, strong tissue specificity, and favorable *in vivo* safety profile, making it especially suitable for repeated dosing and clinical applications^5^. Different AAV serotypes naturally exhibit tissue-specific affinity for organs such as the liver, muscle, and central nervous system. Through genetic engineering techniques like capsid protein modification and peptide tag insertion, these inherent properties can be further enhanced, improving the tissue-targeting capability and immune evasion of AAVs, thereby increasing the efficiency and safety of gene delivery^153^.

AAV enters target cells through receptor-mediated endocytosis, primarily relying on specific cell surface receptors such as heparan sulfate proteoglycans and sialic acids for binding and internalization^154^. Once inside the cell, AAV is transported to the lysosome, where the acidic environment induces a conformational rearrangement of its capsid protein (VP1), exposing the phospholipase A2 domain and facilitating lysosomal escape^155^. Subsequently, the AAV genome enters the nucleus via an importin α/β-mediated nuclear import mechanism^156^. This delivery process is mild and efficient, and AAV typically does not integrate into the host genome. Instead, it primarily exerts its function through episomal expression, thereby minimizing off-target risks. As such, it is well-suited for *in vivo* delivery scenarios requiring short-term gene expression. In contrast, AdV enters cells via Coxsackie and AdV receptor (CAR)-mediated endocytosis and rapidly undergoes capsid disassembly within acidified endosomes, allowing its DNA to directly enter the nucleus and initiate high-level transient expression. AdV offers a relatively large packaging capacity of approximately 36 kb^157^, enabling the simultaneous delivery of multiple CRISPR components, which makes it suitable for constructing multi-target intervention systems. However, its strong immunogenicity can trigger host immune responses, limiting its application in disease models that require repeated dosing^158^. LV typically enters cells through membrane fusion mediated by envelope glycoproteins, though it can also be delivered via endocytosis^159^. Once inside the cell, its RNA genome is reverse transcribed into DNA and integrated into the host genome, enabling stable and sustained gene expression^160^, ^161^. This feature makes LV a valuable vector for long-term gene intervention. For example, Andersen *et al.* demonstrated that using LV-derived nanoparticles to co-deliver RNP and donor template achieved homology-directed repair -mediated gene editing in human hematopoietic stem and progenitor cells, while reducing off-target effects compared to electroporation^162^. However, its genome integration mechanism also poses potential risks of insertional mutagenesis and genomic instability, necessitating careful safety evaluation in clinical translation^5^, ^163^.

Overall, the three viral vectors each have distinct advantages and limitations in terms of membrane translocation mechanisms, expression durability, cargo capacity, and immunogenicity. Among them, AAV remains the preferred platform for *in vivo* CRISPR/Cas delivery due to its well-balanced performance profile. However, sustained expression of CRISPR/Cas components via viral vectors may lead to off-target effects and genomic instability^163^. In addition, high-dose administration of viral vectors carries risks of immunotoxicity, including hepatotoxicity, neurotoxicity, inflammatory responses, and, in rare cases, fatal outcomes^158^, ^164^. For instance, a study utilizing AAV8 vectors for antibody gene delivery found that at doses ≥ 1×10¹² vg/mouse, significant elevations in AST and ALT levels were observed alongside pathological alterations such as hepatocyte vacuolation, confirming the dose-dependent hepatotoxicity^165^. Therefore, clinical translation requires a careful balance between efficacy and potential risks, with comprehensive evaluation of dosing strategies, safety profiles, and long-term effects.

### Non-viral delivery system

Compared to viral vectors, non-viral delivery systems are emerging as a promising alternative for *in vivo* CRISPR/Cas delivery, owing to their superior safety and adaptability^166^. Among them, electroporation and microinjection are classical physical delivery strategies, primarily used in systems such as primary cells, hard-to-transfect tissues, or early-stage embryos. Electroporation enhances membrane permeability through transient electric pulses to facilitate the entry of CRISPR/Cas components into the cytoplasm, and is applicable to a variety of mammalian cells, though it carries a risk of cytotoxicity^167^, ^168^. Microinjection enables precise, single-cell-level delivery and is widely used in embryo editing^169^. While these methods offer high delivery efficiency, their complexity, invasiveness, and limited scalability constrain their broader application in *in vivo* settings^170^. As a result, recent research has increasingly focused on nanoparticle-based chemical delivery platforms, aiming to achieve efficient and targeted CRISPR/Cas transport *in vivo* while maintaining a favorable safety profile.

Among various non-viral vectors, lipid nanoparticles (LNPs) are the most advanced and have been widely applied in human genome editing and preclinical studies^171^, ^172^. LNPs are primarily composed of phospholipids, cholesterol, and ionizable lipids^173^, ^174^, exhibiting excellent biocompatibility and membrane fusion capabilities. They efficiently encapsulate CRISPR/Cas nucleic acids and protein complexes, enabling targeted delivery to specific tissues. For instance, LNP-based CRISPR-Cas9 therapeutic systems can achieve 70-80% gene editing efficiency *in vivo*, resulting in significant tumor growth inhibition of up to 80% in multiple mouse models^175^. However, their main limitations include relatively low cellular uptake efficiency and a tendency to accumulate in filtration organs such as the liver, spleen, and lungs, which can compromise delivery efficiency to other tissues^5^. Currently, LNPs are mainly used in Cas9-mediated gene editing therapies^175^, ^176^, but some studies have extended their application to the field of *in vivo* CRISPR/Cas12a imaging. For example, Li *et al.* developed the “Reset” system, which delivers Cas12a and fluorescent probes via LNPs to achieve dynamic imaging of miRNA-21 in living cells^132^. In the future, LNPs can be functionalized with specific ligands on their surface to enable precise recognition of target cells such as tumors. Combined with fluorescent or multimodal imaging probes, this approach could further enhance the signal-to-noise ratio and delivery efficiency of CRISPR/Cas systems *in vivo*, expanding their potential applications in visualized diagnosis and therapy.

As a representative class of inorganic nanomaterials, AuNPs have become one of the most widely used carriers in CRISPR/Cas-based *in vivo* imaging due to their excellent surface modifiability, unique optical properties, and strong affinity for nucleic acids^142^. The surface of AuNPs can be densely functionalized via thiol groups to anchor sgRNAs, oligonucleotides, and photosensitive dyes, thereby endowing them with multifunctional capabilities including multivalent binding, targeted recognition, and signal responsiveness^142^. In CRISPR-based *in vivo* imaging systems, AuNPs not only efficiently load CRISPR components and facilitate transmembrane delivery, but also possess the ability to quench background signals and enhance probe activity. For example, disulfide-rich oligonucleotides can form dense arrays on the surface of AuNPs, effectively quenching fluorescence signals prior to delivery. Once inside the cell, the reducing environment triggers their release, thereby significantly improving the signal-to-noise ratio and spatial resolution of imaging. Yuan *et al.* developed a CRISPR/Cas12a nanoplatform based on AuNPs, which successfully achieved real-time imaging of specific microRNAs in living cells, demonstrating the great potential of AuNPs in constructing CRISPR nanorobots and regulating dynamic biological processes^49^. In addition, another type of inorganic nanomaterial, silica nanoparticles, has also been applied to the *in vivo* delivery of CRISPR/Cas systems. The InCas platform, constructed based on silica nanoparticles, successfully achieved the visualization of two miRNAs in mice, further expanding the application boundaries of inorganic nanomaterials in CRISPR imaging platforms^150^.

Polymeric organic nanoparticles, such as polyethylenimine (PEI) and poly (lactic-co-glycolic acid) (PLGA), also serve as important delivery vehicles for CRISPR/Cas systems. PEI, which carries a positive charge, can form stable nanocomplexes with negatively charged CRISPR nucleic acid components through electrostatic interactions, and release the active cargo in specific microenvironments via stimuli-responsive mechanisms^177^. For CRISPR/Cas applications requiring gene editing or target imaging within defined spatial and temporal windows, these carriers offer more refined and controllable delivery strategies.

In recent years, DNA nanomaterials have demonstrated unique advantages in the delivery and imaging of CRISPR/Cas systems due to their highly controllable structures, sequence programmability, and excellent biocompatibility^178^, ^179^. DNA nanostructures can precisely regulate their size, spatial configuration, and surface functional groups, enabling the coordinated loading and efficient delivery of multiple components. This high degree of modularity makes them ideal carriers for constructing multifunctional nanoplatforms. For example, researchers have successfully utilized DNA origami technology to build carriers that achieve precise *in vivo* gene editing with the CRISPR/Cas9 system, providing new strategies for gene therapy and molecular diagnostics^180^. Moreover, DNA nanomaterials possess good biodegradability and low toxicity of their degradation products, laying a solid foundation for clinical translation.

Meanwhile, extracellular vesicles (EVs), as natural bio-nanocarriers, have emerged as a promising platform for CRISPR/Cas delivery due to their low immunogenicity, high biocompatibility, and intrinsic transmembrane transport capability^181^. Through engineering modifications, EVs can efficiently load Cas9 and its ribonucleoprotein (RNP) complexes and achieve tissue-specific targeting via membrane-associated native proteins^182^, ^183^. To date, multiple studies have successfully utilized EVs for *in vivo* CRISPR/Cas9 delivery to traditionally hard-to-transfect tissues, including the liver, cardiovascular system, and nervous system^184^-^186^. By incorporating fluorescently labeled RNA or proteins, these systems enable real-time imaging to assess delivery efficiency and targeting specificity. Furthermore, the functional versatility of EVs can be significantly augmented through rational surface engineering strategies. For example, Wang *et al.* developed a valency-controlled tetrahedral DNA nanostructure equipped with DNA aptamers, which was anchored onto the EV membrane via cholesterol modification to achieve cell-specific targeting^187^. This series of advances has laid a solid foundation for the development of efficient and precise CRISPR delivery systems based on EVs.

In summary, the five types of nanoplatforms each possess distinct advantages in the *in vivo* delivery of CRISPR/Cas systems, driving non-viral delivery strategies toward higher efficiency, lower toxicity, and enhanced targeting capability. In the future, through the synergistic use of multiple materials, it is expected to develop CRISPR/Cas *in vivo* visualization delivery systems with richer functionalities and more precise spatiotemporal control.

### Practical challenges and strategies for *in vivo* delivery

The successful delivery of CRISPR/Cas systems to specific organs within a living organism for high signal-to-noise ratio imaging faces significant challenges from multiple physiological barriers, including stability, immunogenicity, and tissue penetration. Naked CRISPR components (e.g., mRNA, sgRNA, RNP) are highly susceptible to nuclease degradation in the systemic circulation and rapid renal clearance, which limits their effective delivery^5^. Although non-viral nanocarriers, such as lipid nanoparticles, can provide protection and enhance stability through encapsulation^171^, they themselves are readily recognized and cleared by the mononuclear phagocyte system (MPS) in the liver and spleen. This leads to non-specific accumulation outside the target organs (particularly the liver) and a shortened circulation half-life^5^, ^171^. To address this, strategies such as PEGylation to create 'stealth' carriers and the design of smart, microenvironment-responsive delivery systems are being actively developed to achieve controlled release within the target tissues.

Beyond the inherent stability of the delivery vehicle itself, immunogenicity is another critical issue that cannot be overlooked. Viral vectors can trigger potent humoral and cellular immune responses, which not only compromise the efficacy of repeated administrations but also carry serious clinical risks such as hepatotoxicity^158^. Non-viral vectors, particularly cationic lipids and polymers, also pose a risk of activating innate immune pathways^171^, ^177^. Most importantly, recent studies have shown that even bacterially derived Cas9 proteins (e.g., saCas9) endogenously expressed after AAV delivery can have their specific T-cell epitopes presented via the host cell's HLA class I molecules. This can activate CD8+ T cells and lead to the clearance of gene-edited target cells^188^. This finding profoundly reveals the immunological hurdles for the long-term application of CRISPR technology and underscores the urgency of developing low-immunogenicity Cas proteins.

The ultimate goal of overcoming these barriers is to achieve high-precision imaging of specific organs or pathological tissues. This highly depends on the application of active targeting delivery strategies. For instance, leveraging the natural tropism of LNPs and certain AAV serotypes (e.g., AAV8, AAV9) for the hepatic MPS, or further modifying them with ligands like galactose to target the asialoglycoprotein receptor on hepatocytes, can efficiently achieve specific liver imaging^153^. For tumors, it is necessary to display tumor-targeting peptides (e.g., RGD peptide) or specific aptamers on the carrier surface to achieve enrichment via receptor-ligand mediated endocytosis^10^. For the brain, an organ tightly protected by the blood-brain barrier (BBB), the strategy shifts towards designing 'transcytosis-enabled' carriers that target specific transporters (e.g., transferrin receptor) on BBB endothelial cells, thereby 'hijacking' endogenous mechanisms to deliver CRISPR components into the brain parenchyma^5^.

In summary, the future development of CRISPR/Cas *in vivo* imaging technology will inevitably rely on 'intelligent' delivery systems that integrate multiple functions. These systems need to synergistically achieve long circulation, immune evasion, specific targeting, and controlled release to navigate precisely through the complex physiological environment, ultimately providing a powerful visualization tool for precision medicine.

## Conclusion and Future Perspectives

The CRISPR/Cas system provides molecular imaging with powerful targeting and dynamic monitoring capabilities, reshaping the foundation of *in vivo* nucleic acid visualization. In recent years, fluorescent probes developed based on this system have steadily overcome the limitations of traditional techniques, ranging from fluorescent protein fusions and high-performance dye labeling to smart responsive mechanisms, multifunctional nanomaterials, and multimodal imaging platforms. These advancements have enabled high-precision observation of gene expression and transcription dynamics within cells, while also offering new tools for disease biomarker detection, targeted delivery, and precise therapy.

However, to fully realize the application of CRISPR/Cas imaging technology in both basic research and clinical settings, several key challenges must still be overcome. First, existing fluorescent probes have limitations in tissue penetration depth, biocompatibility, and photostability, especially in complex microenvironments for *in vivo* imaging. Second, balancing fluorescence signal intensity with target specificity remains an unresolved challenge. While enhancing signal strength is crucial for detecting low-abundance targets, excessive amplification often compromises specificity by increasing nonspecific binding or background fluorescence. Future advances will rely on the design of amplification systems capable of maximizing signal-to-noise ratio without sacrificing molecular precision. Moreover, the safety, targeting capability, and coordinated expression of multiple components in delivery systems remain major bottlenecks restricting the widespread *in vivo* use of CRISPR/Cas imaging technology.

Looking ahead, the development of fluorescent probes for *in vivo* imaging based on the CRISPR/Cas system will focus on several key directions: first, designing smaller and highly programmable fluorescent systems to meet the demands of high-precision imaging at the single-cell and even subcellular levels; second, integrating artificial intelligence with protein engineering to accelerate the rational design and high-throughput screening of novel probe proteins and ligands; third, advancing smarter gating strategies with greater precision and environmental adaptability to enable efficient signal capture and precise regulation under complex pathological conditions; finally, deepening the integration of CRISPR systems with multimodal imaging technologies (such as PET/MRI and photoacoustic imaging) to establish multifunctional platforms that combine diagnosis and therapy.

In summary, as a key technology for visualizing genetic information, the continuous evolution of CRISPR fluorescent probes will not only greatly enrich the foundational toolbox of life sciences but also play a pivotal role in early disease screening, lesion localization, and therapeutic evaluation. This progress is set to drive innovations in precision medicine and bio-diagnostics, opening new avenues for their application.

## Figures and Tables

**Table 1:** Summary of representative CRISPR/Cas systems for biological imaging.

System	Target	Size	PAM/PFS Requirements	Imaging Advantages	Imaging Limitations
Cas9 / dCas9^24^, ^30^	dsDNA	~1368 aa	NGG PAM	Mature technology, high targeting precision; dCas9 allows “label without cutting” live-cell imaging	Large protein size hinders viral delivery; limited signal strength for non-repetitive loci.
Cas12 / dCas12^21^, ^40^	dsDNA,ssDNA	~1300 aa	T-rich PAM (5'-TTTV-3') (V=A/C/G)	Enables signal amplification for highly sensitive visualization; potential for multiplex readout	Trans-cleavage activity unstable in cells.
Cas13 / dCas13^50^	ssRNA	~1200 aa	PFS-dependent (typically non-G)	Direct, reversible, and dynamic RNA tracking in living cells	Signal intensity sometimes weak; activated ssRNA trans-cleavage can be cytotoxic, necessitating dCas13 for safe bioimaging.
Cas14 (Cas12f)^60^	dsDNA,ssDNA	400~700 aa	Minimal or no PAM requirement (5′-T/C preferred)	Small molecular weight, facilitating *in vivo* delivery.	Has not yet been experimentally applied in bioimaging.

**Table 2:** Performance Comparison of CRISPR/Cas Fluorescent Imaging Probes

Probe Category	Sub-type / Strategy	Brightness & Sensitivity	Signal-to-Noise Ratio (SNR)	Photostability	Cytotoxicity	Key Applications & Notes
Fluorescent Protein (FP) Probes	Conventional FPs (e.g., EGFP, YFP)	Medium	Low-Medium	Medium	Low (genetically encoded)	• Long-term live-cell imaging• Multi-color labeling (e.g., CRISPRainbow)
	Signal Amplified Systems (e.g., SunTag, MoonTag)	High	Medium	Medium (depends on FP)	Low (but larger genetic construct)	• Imaging low-abundance nucleic acids• Multi-target tracking
	Split-FP Systems	Low-Medium	Very High	Medium	Low	• Visualizing molecular interactions• High-contrast imaging due to switch-like activation
	Optimized FPs (e.g., mNeonGreen, StayGold, pHuji)	High	Medium-High	High	Low	• Harsh environments (e.g., acidic organelles)• Long-term, high-fidelity imaging
Synthetic Fluorescent Dyes	HaloTag/SNAP-tag coupled dyes (e.g., JF dyes, Alexa Fluor)	Very High	High	Very High	Low-Medium (depends on delivery)	• High-sensitivity detection• High photostability for long tracking
	RNA Aptamer-Ligand (e.g., Spinach/DFHBI, Mango/TO1)	Medium	High (activation upon binding)	Medium	Low (small molecule ligand)	• Real-time RNA imaging• Background suppression via turn-on fluorescence
	Covalent sgRNA labeling (e.g., LiveFISH)	High	High	High (depends on dye)	Medium (requires RNP delivery)	• Simple, universal strategy• High signal consistency for screening
Smart Gated Probes	Photoactivatable	High (after activation)	Very High	High	Low (but UV light can be toxic)	• Precise spatiotemporal initiation of imaging• "Caged" probes for minimal background
	Nucleic Acid-Responsive	Medium-High	High	High	Low (high biocompatibility)	• Sensing intracellular metabolites• Logic-gated activation
	Logic-Gate Probes	Medium-High	Very High	High	Low	• High specificity for complex biomarkers• Reduces false positives (e.g., AND gates)
Nanomaterial-Based Probes	Gold Nanoparticles (AuNPs)	High (signal quenching/release)	High	Extremely High	Medium-High (potential for accumulation)	• Controlled release platforms• Enhanced cellular delivery• Multimodal potential
	Quantum Dots (QDs)	Extremely High	High	Extremely High	Medium-High (blinking, potential toxicity)	• High-throughput, sensitive imaging• Multiplexing due to narrow emission spectra
Multimodal Fusion Probes	Cas9-based Theranostic	High	Medium-High	High	Medium-High (combined therapy)	• Image-guided gene editing• Combined therapy (e.g., photodynamic)
	Cas13-based Theranostic	High	Medium-High	High	Medium	• Real-time RNA imaging and intervention• High spatiotemporal control

**Table 3:** Summary of Smart Gated Probes for CRISPR/Cas-Mediated Bioimaging.

Probe Type	Representative Study	Core Mechanism and Features	Application Advantages
Photoactivatable Probe	LAC12a system^115^	Incorporation of photocaged group into crRNA hairpin to enable blue-light controlled Cas12a activation	Non-invasive, dynamically controllable, high signal-to-noise
	MF@Cas12a/crRNA@RS^124^	Construction of a dual-component system with UCNPs/photocaged hairpins and MnO₂/Cas12a complex for NIR-triggered activation and signal amplification	Deep tissue imaging; signal amplification
	UCNP@MnO2@CRISPR/Cas12a^48^	Spatiotemporal control, enhanced cleavage activity, sensitive detection via HCR	Deep tissue imaging; signal amplification
Nucleic Acid-Gated Probe	NIR Photoactivatable Aptamer-CRISPR Nanodevice (Apt-CNs)^14^	Aptamer unlocks upon binding target molecule, NIR light further regulates structure for spatiotemporal IFN-γ imaging	High specificity; no external stimuli required
	RESET-Cas12a^132^	Hairpin structure locks key sequence; target recognition triggers unlocking and CRISPR/Cas activation	Accurate response; structurally simple; reversible control
Logic-Gated Probe	InCasApt^150^	Multi-input AND logic combining aptamer recognition and photosensitizer activation for synergistic theranostics	High specificity; reduced off-target activation; achieve theranostics integration

**Table 4:** Overview of Representative Multimodal CRISPR Probe Platforms Based on Different Cas Proteins.

Cas System	Probe Platform Name	Probe Composition	Functional Modules	Imaging Modality	Therapeutic Modality	Application Highlights
Cas9	TCPH^10^	Cas9 + AIE photosensitizer + PEI/HA coating	Gene editing coupled with photosensitive activation	Fluorescence / NIR	Photodynamic therapy combined with immune activation	Precise tumor-site intervention with real-time therapeutic visualization
Cas9	PPMC@CM^189^	Cas9-PDL-1 system + MnO2 nanoparticles	PD-L1 gene disruption integrated with hypoxia relief and GSH depletion	MRI	Chemodynamic therapy synergized with immune checkpoint blockade	Biomimetic platform enabling MRI-guided synergistic CDT and CRISPR-enhanced immunotherapy
Cas12a	CRISPR/Cas 12a-Fe3O4@mSiO2^151^	Cas12a+Fe₃O₄@mSiO₂@methylene blue+G-quadruplex/hemin complex	miRNA-triggered PER coupled with Cas12a activation for dual-mode signal amplification	SERS/colorimetric readout	None	Ultrasensitive detection of miRNA-21 using a dual-modality platform integrating SERS and colorimetry.
Cas13a	InCasApt^150^	Cas13+UCNPs+light-controlled switch	Fluorescence-guided RNA imaging and light-triggered photodynamic therapy	Fluorescence / NIR	Photodynamic therapy	Spatiotemporally controllable imaging in live cells and deep tissues, suitable for RNA-targeted applications

**Table 5:** Summary of CRISPR/Cas delivery systems and their applications**.**

Delivery System		Cargo Format	Description	Visualization	Delivery targets	Advantage	Limitation	Reference
Virus Delivery System	Adeno-associated virus (AAV)	Cas + sgRNA expression cassette	Utilizes AAV to deliver CRISPR components.	Nucleus targeting, using FPs for target visualization and avoiding off-target risks	Somatic & germline cells	Low immunogenicity; not integrated into the genome	Limited packaging capacity (∼4.7 kb); potential biosafety concerns associated with viral vectors.	190, 191
	Lentivirus (LV)	Cas + sgRNA expression cassette (integrating DNA)	Utilizes LV to deliver CRISPR components with genomic integration for sustained expression.	Nucleus targeting, using FPs for target visualization and avoiding off-target risks	Somatic & germline cells	Long-term expression; suitable for stable cell line generation.	Integrates into the host genome, posing a risk of insertional mutagenesis; limited packaging capacity (~8 kb); potential biosafety concerns associated with viral vectors.	192
	Adenovirus (AdV)	Cas + sgRNA expression cassette	Utilizes AdV to deliver CRISPR components for transient, high-level expression.	Nucleus targeting, using FPs for target visualization and avoiding off-target risks	Somatic	Large packaging capacity (~36 kb); low risk of insertional mutagenesis	Transient expression limits long-term editing; strong immunogenicity may cause inflammation.	193
Non-Viral Delivery Systems	*In vitro* Electroporation	Cas protein& sgRNA	Temporarily opens cell membranes with electric pulses to deliver CRISPR components.	Can combine with fluorescent tags on cargo for tracking efficiency.	Cell lines, primary cells	High delivery efficiency; suitable for large cargos including RNP complexes	Cell viability may be reduced due to electrical stress	194
	*In vivo* Electroporation		Delivering CRISPR components to the appropriate tissue by applying the pulse directly to the tissue of the organism		Living tissue	Capable of targeting specific tissues for precise therapy.	Limited applicability *in vivo*; requires specialized equipment.	195
	*Ex vivo* electroporation		CRISPR components are delivered into cells *ex vivo* by electroporation, and the modified cells are then reinfused into the body.		T cells, Hematopoietic stem cells	Enables precise control; offers improved safety and therapeutic efficacy.	High technical complexity and demanding experimental conditions.	196
	Microinjection	CRISPR/Cas RNP	Direct physical injection of CRISPR components into individual cells or embryos under microscopy.	Allows real-time visualization during injection.	Zygotes, embryos, single cells	Precise delivery; minimal off-target effects; suitable for single-cell editing.	Labor-intensive; requires skilled operators and specialized equipment; not suitable for large-scale applications.	197-199
	Lipid Nanoparticles (LNPs)	CRISPR/Cas RNP	Utilize lipid-based nanoparticles to encapsulate and deliver CRISPR components efficiently into cells.	Can be combined with fluorescent labels for tracking delivery and uptake.	Somatic	High delivery efficiency; low immunogenicity; capable of delivering large cargos.	Potential off-target delivery; possible toxicity depending on formulation; limited tissue targeting without modification.	5, 200
	Gold Nanoparticles (AuNPs)	CRISPR/Cas RNP& Plasmids	AUNPs carry CRISPR components through functionalized surfaces for better uptake.	Can be conjugated with fluorescent probes for tracking delivery and localization.	Somatic, Tumor cells	High surface area for functionalization; good biocompatibility.	Potential cytotoxicity at high concentrations; possible aggregation; limited tissue penetration without targeting modifications.	49, 201
	Silica Nanoparticles	CRISPR/Cas RNP	Utilize functionalized silica nanoparticles to load and deliver CRISPR components into cells.	Lipid modification followed by loading of imaging-related probes	Somatic, Tumor cells	High stability; large surface area; protects cargo from degradation.	Possible slow release; requires surface functionalization for targeting.	150, 202
	PEI	CRISPR/Cas RNP& Plasmids	A cationic polymer that forms electrostatic complexes with CRISPR components for intracellular delivery.	Labeling genetically modified cells with fluorescent markers	Somatic, Tumor cells	Easy to synthesize; low cost; efficient cellular uptake.	High cytotoxicity at high concentrations; limited biodegradability	203
	PLGA	CRISPR/Cas RNP& Plasmids	Biodegradable polymeric nanoparticles that encapsulate CRISPR components for controlled intracellular delivery.	Can be co-loaded with fluorescent dyes for imaging and tracking.	Somatic, Tumor cells	Biocompatible and biodegradable; sustained and controlled release	Limited transfection efficiency without surface modification; slow release may delay gene editing activity.	204, 205
	DNA Nanomaterials	CRISPR/Cas RNP& Plasmids	Engineered DNA nanostructures (e.g., tetrahedrons, origami) are used to encapsulate or anchor CRISPR components for targeted delivery.	Can be integrated with fluorophores or aptamer-based imaging modules.	Somatic, Tumor cells	High programmability; precise structural control; biocompatibility	Susceptible to nuclease degradation; relatively low stability *in vivo*	180, 187
	Extracellular Vesicles (EVs)	CRISPR/Cas RNP& Plasmids	Natural membrane-bound vesicles secreted by cells, engineered to carry CRISPR components into target cells.	Can be labeled with membrane dyes or engineered with fluorescent tags.	Somatic, Tumor cells	Low immunogenicity; high biocompatibility; endogenous targeting potential.	Complex isolation and purification; potential off-target distribution.	186

## Abbreviations

AAV: adeno-associated virus
AdV: adenovirus
AuNPs: gold nanoparticles
BAP-tag: biotin acceptor peptide
CRISPR: Clustered Regularly Interspaced Short Palindromic Repeats
DFHBI: 3,5-difluoro-4-hydroxybenzylidene imidazolinone
DMNB: 4,5-dimethoxy-2-nitrobenzyl
dsDNA: double-stranded DNA
EVs: extracellular vesicles
FISH: fluorescence *in situ* hybridization
FPs: fluorescent proteins
GFP: green fluorescent protein
HCR: hybridization chain reaction
JF: Janelia Fluor
LV: lentivirus
MB: molecular beacon
MCP: MS2 coat proteins
MTS: molecular beacon target sequence
NIR: near-infrared
PAM: protospacer adjacent motif
PEI: polyethylenimine
PLGA: poly (lactic-co-glycolic acid)
QDs: quantum dots
RNP: ribonucleoprotein
sgRNA: single-guide RNA
SNR: signal-to-noise ratio
ssDNA: single-stranded DNA
UCNPs: upconversion nanoparticles
UV: ultraviolet
